# A de novo variant in *ADGRL2* suggests a novel mechanism underlying the previously undescribed association of extreme microcephaly with severely reduced sulcation and rhombencephalosynapsis

**DOI:** 10.1186/s40478-018-0610-5

**Published:** 2018-10-19

**Authors:** Myriam Vezain, Matthieu Lecuyer, Marina Rubio, Valérie Dupé, Leslie Ratié, Véronique David, Laurent Pasquier, Sylvie Odent, Sophie Coutant, Isabelle Tournier, Laetitia Trestard, Homa Adle-Biassette, Denis Vivien, Thierry Frébourg, Bruno J Gonzalez, Annie Laquerrière, Pascale Saugier-Veber

**Affiliations:** 1 Normandie Univ, UNIROUEN, Inserm U1245, Normandy Centre for Genomic and Personalized Medicine, F 76000 Rouen, France; 2 Normandie Univ, UNICAEN, Inserm U1237, F 14000 Caen, France; 3 Rennes1 University, Faculty of Medicine, UMR6290 CNRS IGDR, F 35000 Rennes, France; 4 Department of Genetics, Rennes University Hospital, F 35000 Rennes, France; 5 Belvedere Hospital, Department of Genetics, F 76130 Mont-Saint-Aignan, France; 6 Lariboisière Hospital, APHP, Department of Pathology, F 75000 Paris, France; 7 Paris Diderot University, Sorbonne Paris Cité, PROTECT INSERM, F 75019 Paris, France; 8 Department of Genetics, Normandy Centre for Genomic and Personalized Medicine, Rouen University Hospital, F 76000 Rouen, France; 9 Department of Pathology, Rouen University Hospital, F 76000 Rouen, France

**Keywords:** ADGRL2, LPHN2, Adhesion-GPCR, Alpha-latrotoxin, Human extreme microcephaly, Rhombencephalosynapsis

## Abstract

Extreme microcephaly and rhombencephalosynapsis represent unusual pathological conditions, each of which occurs in isolation or in association with various other cerebral and or extracerebral anomalies. Unlike microcephaly for which several disease-causing genes have been identified with different modes of inheritance, the molecular bases of rhombencephalosynapsis remain unknown and rhombencephalosynapsis presents mainly as a sporadic condition consistent with de novo dominant variations. We report for the first time the association of extreme microcephaly with almost no sulcation and rhombencephalosynapsis in a fœtus for which comparative patient-parent exome sequencing strategy revealed a heterozygous de novo missense variant in the *ADGRL2* gene. *ADGRL2* encodes latrophilin 2, an adhesion G-protein-coupled receptor whose exogenous ligand is α-latrotoxin. Adgrl2 immunohistochemistry and in situ hybridization revealed expression in the telencephalon, mesencephalon and rhombencephalon of mouse and chicken embryos. In human brain embryos and fœtuses, Adgrl2 immunoreactivity was observed in the hemispheric and cerebellar germinal zones, the cortical plate, basal ganglia, pons and cerebellar cortex. Microfluorimetry experiments evaluating intracellular calcium release in response to α-latrotoxin binding showed significantly reduced cytosolic calcium release in the fœtus amniocytes vs amniocytes from age-matched control fœtuses and in HeLa cells transfected with mutant ADGRL2 cDNA vs wild-type construct. Embryonic lethality was also observed in constitutive *Adgrl2*^−/−^ mice. In *Adgrl2*^+/−^ mice, MRI studies revealed microcephaly and vermis hypoplasia. Cell adhesion and wound healing assays demonstrated that the variation increased cell adhesion properties and reduced cell motility. Furthermore, HeLa cells overexpressing mutant *ADGRL2* displayed a highly developed cytoplasmic F-actin network related to cytoskeletal dynamic modulation. *ADGRL2* is the first gene identified as being responsible for extreme microcephaly with rhombencephalosynapsis. Increased cell adhesion, reduced cell motility and cytoskeletal dynamic alterations induced by the variant therefore represent a new mechanism responsible for microcephaly.

**Electronic supplementary material:**

The online version of this article (10.1186/s40478-018-0610-5) contains supplementary material, which is available to authorized users.

## Introduction

At the end of the 4th post-conception week (PCW), the neural tube closes and immediately undergoes drastic changes, which consist in the setting of several events regulated by multiple, often redundant, signalling pathways leading to anteroposterior and dorsoventral polarity and emergence of four curvatures that demarcate the primary cerebral vesicles—the prosencephalon, the mesencephalon, pons and myelencephalon—from the spinal cord. Concomitantly, other key events come into play to allow the proper growth, folding and differentiation of all brain structures and particularly of the cerebral cortex; these events are schematically divided into three main stages encompassing cell proliferation with expansion of the progenitor population, neuronal migration and post-migration developmental processes. The critical role of these events, which are necessary for appropriate development and function of the human six-layered cortex, is reflected by the wide range of disease phenotypes arising from their disruption, the most severe of them being polymicrogyria, lissencephaly, microcephaly and microlissencephaly.

Lissencephalies are usually single-gene disorders that affect neuronal migration during cortical development; polymicrogyria, which has been associated with genetic and environmental causes, is still often considered as secondary to abnormal post-migration development [9, 21, 22, 33]. Microlissencephaly is a rare condition characterized by severe congenital microcephaly with absent sulci and gyri with either a thinned or thickened cortical plate. Similar to polymicrogyria, microcephaly and microlissencephaly may be due to both genetic and environmental causes, the latter including infections, toxic insults (notably antiepileptic drugs, opioids or cocaine) and prenatal alcohol exposure. Genetic causes are multiple and result from abnormal neuronal proliferation or survival associated with defective neuronal migration [5, 21]. Whatever the cause, lissencephaly and microlissencephaly may be observed alone or in combination with various brainstem or cerebellar lesions.

Among the diverse cerebellar developmental abnormalities, rhombencephalosynapsis (RES) is an extremely rare malformation initially described by Obersteiner as complete or partial vermis agenesis with fusion of the cerebellar hemispheres and apposition or fusion of the deep cerebellar nuclei [55]. RES is thought to occur early during embryogenesis, between the 5^th^ and 7^th^ PCW, but its pathophysiological mechanism remains a matter of debate, considered by some authors as resulting from a fusion and by others from a non-separation of cerebellar hemispheres over an absent or severely hypoplastic vermis [8, 32, 56]. RES occurs in a vast majority of cases as a sporadic condition consistent with de novo dominant variations, and to date, exceedingly rare syndromic forms have been described and comprise Gomez-Lopez-Hernandez syndrome (MIM#601853), Fanconi anaemia complementation group B (MIM#300514) and autosomal recessive (MIM#276950) or X-linked (MIM#314390) inherited condition designated VACTERL-H [19]. In sporadic forms, RES occurs in isolation or in combination with other central nervous system (CNS) and extra-CNS malformations; it has been described in association with mesencephalic lesions such as atresia forking of the aqueduct of Sylvius and fusion of the colliculi. Associated supratentorial lesions have also been reported, consisting in agenesis of the corpus callosum, atresia of the 3^rd^ ventricle, holoprosencephaly and neural tube closure defects [56]. So far, however, the association of severe microcephaly with RES has never been reported to our knowledge.

Using comparative patient-parents exome sequencing strategy, a powerful method to detect de novo pathogenic variants involved in human Mendelian genetic diseases [52, 53], we identified the first molecular basis of this association of extreme microcephaly with severely reduced sulcation with RES in a fœtus, a deleterious variant in the *ADGRL2* gene, which encodes an adhesion G-Protein-Coupled Receptor (GPCR). Mechanistic and functional characterization of the variant provides compelling evidence that this deleterious variant causes early human developmental defects involving both supratentorial and infratentorial structures.

## Materials and methods

### Whole exome sequencing

The parents provided written informed consent for Whole Exome Sequencing (WES). High quality genomic DNA was extracted from the peripheral blood of the fœtus and her parents using QIAamp DNA Blood Midi Kit (Qiagen, Courtabœuf, France) and QuickGene DNA Whole Blood Kit L (Kurabo, Japan), respectively, according to the manufacturer’s instructions. Approximately 3 μg was sheared with a Covaris E220 DNA Sonicator (Covaris, Inc., Woburn, MA, USA) and coding regions captured using a SureSelectXT Human All Exon V2 kit (Agilent Technologies, Santa Clara, CA, USA) according to the manufacturer’s instructions. The enriched libraries were sequenced on a Genome Analyzer IIx (GAIIx, Illumina, Inc., San Diego, CA, USA) with 76 bp paired-end reads. Image analysis and base calling were performed by Real-Time Analysis (RTA 1.10) and CASAVA software (v1.8, Illumina, Inc.). Reads were mapped to the human reference sequence (GRCh37, Hg19) with the Burrows-Wheeler Aligner (BWA v.0.6.2). Read duplicates were marked with Picard tools, local realignments around indels, base-quality-score recalibration and variant calling were performed with the Genome Analysis Toolkit (GATK 2.5). Single-nucleotide variants and small indels were identified with the GATK UnifiedGenotyper and were filtered according to the Broad Institute’s best-practice guidelines (Additional file 1: Table S1). Variants were then annotated with ANNOVAR (version 2012). Filtration of unknown variations and differential exome analysis were achieved using the Exome Variation Analyzer (EVA 2.0), our in-house software [16]. To evaluate its pathogenic potential, the *ADGRL2* DNA sequence alteration was analysed in the following web-based programs: MutationTaster [60], SIFT [40] and PROVEAN [15].

### Sanger sequencing analysis

The 20 *ADGRL2* exons, 100 bp exon-intron boundaries and UTRs were PCR amplified from 50 to 100 ng of genomic DNA extracted from peripheral blood (exome trio) and from fœtal tissues coming from the Department of Genetics, Rennes University Hospital. These DNA samples were first amplified using the Whole Genome Amplification GenomePlex2 kit (Sigma-Aldrich, St Louis, MO, USA). Sanger sequencing of these fragments was performed using the BigDye® Terminator v3.1 Cycle sequencing Kit (Applied Biosystems, Courtabœuf, France). Sequencing reactions were migrated on a 3100xl Genetic Analyzer (Applied Biosystems) and analysed using the Sequencing analysis software 5.2.0 (Applied Biosystems). PCR and sequencing primers are available upon request.

### *Adgrl2* mouse and chicken in situ hybridization

Chick (*Gallus gallus*) or mouse (C57Bl6) embryos were fixed overnight at 4 °C in 4% paraformaldehyde (PFA), rinsed and processed for whole-mount RNA in situ hybridization. Chick embryos were staged according to Hamburger and Hamilton (HH) [27]. For the hybridization step, embryos were permeabilized 5 min in proteinase K solution (10 μg/ml), then fixed for 20 min in 4% PFA/0,2% Glutaraldehyde. After several washes in PBT, the embryos were incubated in a prehybridization solution (50% formamide, 5× SSC pH 4.5, 2% SDS, 2% blocking reagent (Roche, Meylan, France), 250 μg/ml tRNA, 100 μg/ml Heparin) at 65 °C before the addition of 10 μg/ml of the *Adgrl2* probe, and overnight incubation at 65 °C. Probes were generated by PCR, subcloned in pCRII-TOPO® (Invitrogen, Saint Aubin, France), and used to transcribe the digoxigenin (DIG)-labelled antisense RNA probes. After incubation, embryos were washed 4 times for 30 min with a solution containing 50% formamide, 2× SSC and 1% SDS at 65 °C, then cooled down to room temperature in 1 M maleic acid buffer containing Tween 20 (MABT) and washed several times. For the antibody step, nonspecific binding was blocked by incubating 2 times for 30 min then 1 h in MABT containing a 2% blocking reagent solution and 20% normal calf serum. AP-conjugated anti-DIG antibody was added at a concentration of 1:3000 and incubated overnight at 4 °C. The embryos were washed 5 times for 1 h in MABT at room temperature, followed by 2 times for 10 min in a solution containing 100 mM NaCl, 100 mM Tris-HCL, 50 mM MgCl2 and 0.1% Tween20 at pH 9.5 (NTMT). The AP-conjugated anti-DIG antibody was detected by a mixture of NBT/BCIP in NTMT, pH 9.5. The reaction was stopped by washing in PBT once the required staining intensity was achieved.

### ADGRL2 immunohistochemical studies in normal human embryos and fœtuses

A series of 3 embryos and 19 fœtuses were selected for this study (collection number DC-2015-2468, cession number AC-2015-2467). Detailed characteristics of the selected cases are presented in Additional file 2: Table S2. Gestational age was estimated according to biometric data, skeletal measurements and histological maturation of the brain and viscera. Six-μm paraffin-embedded sections from whole embryos (6–10 PCW) and from brains and gonads from fœtuses at 13 weeks of gestation (WG) to birth were mounted on coated slides (Superfrost Slides, Thermo Fisher Scientific, Illkirch, France) and dried overnight in a convection oven (37 °C). Induced epitope retrieval was performed by immersion in a citrate buffer solution pH 6 at 95 °C for 1 h. Incubations with the primary antibody ADGRL2 (diluted 1:200, Clinisciences, Nanterre, France) were carried out for 1 h at room temperature using the Benschmark Ultra system (Ventana Medical Systems, Tucson, AZ), the primary antibody being diluted in an antibody diluent reagent solution (Life technologies, Saint Aubin, France). After incubation, slides were processed by the detection kit Ultraview (Ventana Medical Systems). Peroxidase was visualized using the alkaline phosphatase detection kit (Ventana Medical Systems). Slides were rinsed in tap water, counterstained with hæmatoxylin and mounted in mounting medium. Negative controls were obtained by omission of the primary antibody or by the use of other antibodies of known reactivity.

### Cell culture

Amniocytes from control and patient fœtuses were collected by amniocentesis at 19 WG in order to explore chromosomal abnormalities. HeLa and amniocytes cultures were grown as monolayer in T-75 flasks. Cells were incubated in Ham’s F12 nutrient mixture (Gibco, Life Technologies, Saint-Aubin, France) containing 10% fœtal bovine serum (Gibco) and 2 mM L-Glutamin (Sigma-Aldrich) at 37 °C in an atmosphere of 5% CO2.

### Immunoblotting

Amniocytes were washed once with 1× phosphate saline buffer (PBS), trypsinized, centrifuged at 1,500 × rpm for 5 min, and solubilized in 100 μl of RIPA buffer (Thermo Fisher Scientific) with 1× Protease Inhibitor Cocktail (Sigma-Aldrich) and 1× Phosphatase Inhibitor Cocktail (Thermo Fisher Scientific) for 30 min at 4 °C. After 30 min of centrifugation at 14,000×g, 30 μg proteins were separated by denaturing sodium-dodecyl sulphate polyacrylamide gel electrophoresis (10%, SDS-PAGE) and transferred to nitrocellulose membrane (Hybond C-Extra; Amersham Biosciences, Arlington Heights, IL, USA). Blots were blocked for 1 h with 5% skimmed milk in PBS and incubated with anti-ADGRL2 polyclonal antibody (1:500, LifeSpan BioSciences, Seattle, WA, USA) or anti-GAPDH polyclonal antibody (1:1000, Abcam, Cambridge, UK) in 0.05% Tween-PBS (PBST) overnight at 4 °C under gentle agitation. Membranes were washed with PBST, and primary antibody was detected using peroxidase-labelled anti-rabbit or anti-goat antibodies (1:10,000, Jackson Immunoresearch Laboratories, West Grove, PA, USA). Signals were detected with chemiluminescence reagents (Pierce Biotechnology, Rockford, IL, USA) and acquired with a G:BOX (Syngene, Cambridge, UK), monitored by the Gene Snap software (Syngene). The signal intensity in each lane was quantified using the Genetools software (Syngene), and the ratio of ADGRL2 signal vs GAPDH was calculated. All immunoblotting experiments were carried out in triplicate.

### Plasmids and cell transfection

The *Adgrl2* variation was introduced using Quick Change XL Site-Directed Mutagenesis Kit (Agilent Technologies), into previously published pcDCIRL-2 or pcDCIRL-2-GFP expression plasmids containing the full length rat Adgrl2 cDNA [31]. Owing to the fact that rat wild-type Adgrl2 protein contains a phenylalanine (TTC) instead of the leucine (CTC) at position 1262 in humans (same hydrophobic class, Grantham distance 22), we performed a double mutagenesis to introduce the mutant-related histidine (CAC) at position 1262. Wild-type and mutant pcDCIRL-2 expression plasmids (2 μg) were transiently transfected into HeLa cells, using fuGENE 6 transfection reagent (Promega, Madison, WI, USA) according to the manufacturer’s protocol. Cells were plated at 5 × 105 cells/well in 6-well plates in Ham’s F12 medium, and transfections were incubated for 48–72 h to allow Adgrl2 addressing to the plasma membrane. All transfection experiments were carried out in triplicate.

### Microfluorimetry and intracellular calcium measurements

For measurement of whole cell intracellular calcium levels, amniocytes or HeLa cells were grown in 6 well plates on glass coverslips (diameter 30 mm) coated with poly-L-lysine (Sigma-Aldrich). Cultured cells were rinsed in PBS and incubated in Ham’s F12 culture medium containing 10 μM of the calcium probe Fura-2 AM, 0.3% pluronic F-127 (Molecular Probes, Life Technologies, Cergy-Pontoise, France) and 50 μM MK-571 sodium salt hydrate (Sigma Aldrich) for 30 min at 37 °C in the dark. After the loading step, cells were washed twice for 5 min in culture medium, and the coverslips supporting the cells were placed under an inverted fluorescence Leica DM 6000B microscope equipped with a rapid shutter wheel (Rueil-Malmaison, France) and continuously infused with Ham’s F12-EDTA solution. The fluorescent signals associated with calcium-free and calcium-bound Fura-2 were acquired by alternately exciting the cells at 340 and 380 nm. The emitted fluorescence was collected at 510 nm and a ratio of both signals was calculated using the Metamorph software (Roper Scientific, Evry, France). Total recording time was 15 min, and the time interval between two acquisitions was 5 s. After 3 min baseline recording, α-latrotoxin (1 nM, Alomone Labs, Jerusalem, Israel) was added in the calcium-free perfusion medium. To allow dimer formation, α-latrotoxin was prepared in Ham’s F12 calcium free medium 30 min before use. After a 3-min period under drug stimulation, the perfusion medium was replaced by Ham’s F12 containing 1.2 mM calcium. The exogenous ligand α-latrotoxin induces intracellular Ca^2+^ (Ca^2+^_i_) elevation by two mechanisms that are not mutually exclusive, i.e.*,* activation of phospholipase C (PLC) through specific ADGRL receptor activation [4, 42] and the ionophoric properties of α-latrotoxin [3, 30]. Culture media complemented with 4 nM EDTA were used to induce extracellular Ca^2+^ chelation and target intracellular calcium flux resulting from ADGRL receptor activation [71]. When required, cells were incubated during the loading step with 10 μM of the phospholipase C inhibitor U73122 (Sigma Aldrich) [10]. Data were then exported to the biostatistics Prism software (GraphPad Inc., San Diego, CA) and areas under the curve were quantified. For statistical analyses, experiments were performed three times on at least 20 cells per condition.

### Cell cycle analyses in human fœtal ganglionic eminences

Since the germinal zone of the dorsal telencephalon disappears by 24WG, proliferative stem cells were evaluated on lateral ganglionic eminences (LGE), which are known to massively produce interneurons at this stage [50]. LGE were micro-dissected from the paraffin-embedded section passing through the diencephalon from the fœtus, from an age-matched control brain aged 26WG as well as from a fœtus interrupted for microcephaly due to homozygous pathogenic variant in the *MCPH1* gene, NM_024596.3:c.427dup;p.(Thr143Asnfs*5). *MCPH1* variations are responsible for delayed mitosis of cycling progenitors in the germinal zones, and in microlissencephaly brain lesions may also include extreme hypoplasia of the cerebellum and brainstem. The sections were treated using the technique described by Hedley et al. with minor modifications [29]. Briefly, 35-μm sections were dewaxed in xylene and rehydrated in decreasing concentrations of ethanol. Enzymatic digestion was carried out in a 0.5% pepsin PBS solution. The nuclear suspension, adjusted at a final concentration of 10^6^ nuclei/ml were incubated in a 1 mg/ml ribonuclease solution for 30 min at 37 °C and stained with propidium iodide (6 μg/ml in a 40 μM trisodium citrate solution) for 1 h at 4 °C. Flow cytometric analyses of the cell cycle were performed on an XL Beckman Coulter flow cytometer (Coultronics, Hialeah, Florida, USA) with the 488-nm wavelength of an ion argon laser as an excitation source. The cytofluorograph was adjusted to maximal resolution with flow check microspheres (Coultronics) having a coefficient of variation lower than 1.5%. For each sample, 10^4^ to 10^5^ cells were analysed, and the proliferative index (PI) was estimated using the Multicycle software (Coultronics), calculated as the percentage of cells in phases S + G2/M.

### Cytometric analyses of Adgrl2 transfected HeLa cells

HeLa cells were transfected with an empty vector or with plasmids encoding the wild-type or the mutant Adgrl2 cDNA. After a 3-day culture, cells were gently detached from their support using 1 mM EDTA in PBS and processed for cell cycle analysis according to the three steps method of Vindeløv [70]. Single cell suspensions were obtained after incubation in a 3% trypsin PBS buffer solution. Cell suspensions were filtered through a 48-μm pore nylon gauze. Cell count was adjusted to obtain a final concentration of at least 10^4^ cells. The suspensions were mixed with chicken red blood cells (CRBC) used as internal standard, as described by Vindeløv et al. [70]. The concentration was adjusted to obtain a final ratio of CRBC to cultured cells of 1/10. An aliquot of 300 μL of these nuclear suspension samples was centrifuged at 500 g, then resuspended in 500 μl of a staining solution including RNase, propidium iodide and non-ionic detergent Nonidet P40 for 10 min at 4 °C. Evaluation of the cell cycle was performed as described above. For evaluation of cell size and content, 5000 to 6000 cells were incubated in a PBS solution containing 0.3% saponin, 1% bovine serum albumin, 1% RNase and 0.005% propidium iodide. The size corresponding to the light emitted by the cells under exposition to the incident light of the laser (forward scatter) was arbitrarily expressed by the ratio number of cells vs fluorescence intensity on a scale of 1024 channels (AU, arbitrary units). The cellular content corresponding to the more or less heterogeneous cytoplasmic content (granulometry) was evaluated using emitted scattered light of the cell at 90° (side scatter) also expressed by the ratio number of cells vs fluorescence intensity on a scale of 1024 channels.

### Confocal microscopy

To study the addressing of Adgrl2 to the plasma membrane, HeLa cells were grown in 6-well plates on glass coverslips (diameter 10 mm) coated with poly-L-lysin (Sigma-Aldrich). HeLa cells were transfected with 2 μg pcD-empty vectors or with pcD plasmids encoding Wt and mutant rat Adgrl2 (CIRL-2) constructs coupled with a GFP tag using the fuGENE 6 transfection reagent (Promega). After 72 h, glass coverslips were rinsed with PBS and cells were fixed with 4% paraformaldehyde in PBS for 15 min. After 3 gentle washes with PBS, coverslips were mounted in DAPI-containing Vectashield (Vector laboratories, Cambridgeshire, UK) and images were acquired with the Leica laser scanning confocal microscope TCS SP2 AOBS (Leica Microsystems, Wetzlar, Germany).

### Cell-adhesion assays

Cell adhesion assays were performed in triplicate with HeLa cells as previously described [12]. HeLa cells were transfected with 2 μg pcDCIRL-2 Wt or pcDCIRL-2 Mt. coupled or not with a GFP tag using fuGENE 6 transfection reagent (Promega). After 72 h, the living and dead cells were respectively labelled with cell tracker green (Invitrogen) and 7-amino-actinomycin D (7-AAD, Sigma Aldrich). Cells were gently detached using 1 mM EDTA in PBS and 5.10^5^ cells were resuspended in 330 μL of aggregation medium (Ham’s F12 containing 10% FBS, 50 mM Hepes-NaOH, pH 7.4, 10 mM CaCl_2_, and 10 mM MgCl_2_) using a Countess Automated Cell Counter (Invitrogen). Cell suspensions were placed into 0.5-ml polypropylene tubes, leaving a small air bubble between the liquid and the lid and incubated under gentle agitation at 25 °C. Cell aggregation was addressed at T0 and T90 min by removing aliquots, spotting them onto culture slides (BD Falcon), and imaging the aggregates with a Leica DM 6000B microscope. Acquired images were then analysed by quantifying the number and size of aggregates using the Metamorph software (Roper Scientific). The mean aggregation index was calculated using the formula: (total aggregate area / aggregate number)_T90_ − (total aggregate area / aggregate number)_T0_. When required, the aggregation index of the HeLa cells was determined in the presence of 1 nM α-latrotoxin or 3 μM U73122 added in the aggregation medium.

### Wound healing assay

HeLa cells transfected with 2 μg pcD-empty, pcDCIRL-2 Wt or pcDCIRL-2 Mt. plasmids were seeded in 6-well plates at a density of 5 × 10^5^ cells per well. When cells reached confluence, a scratch was performed with a sterile tip to create an artificial wound. Tiff format images were acquired every 6 h during 72 h using an inverted microscope (Leica DM 6000B). Cell migration from the wound edge into the wound space was recorded and a time-course quantification of the scratch width was evaluated using the Metamorph software (Roper Scientific). Cell wound repair was calculated using wound width (expressed in percentage of the initial size).

### Cytoskeletal network immunolabelling

HeLa cells were grown in 6-well plates on glass coverslips (10 mm diameter) coated with poly-L-lysin (Sigma-Aldrich). Cells were transfected with 2 μg pcD-empty or pcD plasmids encoding Wt or Mt. rat Adgrl2 (CIRL-2) using fuGENE 6 transfection reagent (Promega). After 72 h, glass coverslips were rinsed with PBS and fixed 15 min with 4% paraformaldehyde in PBS. HeLa cells were incubated overnight at 4 °C with an anti-acetylated α-tubulin monoclonal antibody (T-9026; Sigma-Aldrich) and Texas Red®-X Phalloidin (Molecular Probes) in the incubation buffer (PBS containing 1% bovine serum albumin (BSA) and 3% Triton X-100). Cells were rinsed twice with PBS for 20 min and incubated with the same incubation buffer containing the appropriate secondary antibody. Nuclei were visualized by incubating the cells for 5 min with a PBS solution containing 1 μg/mL Hœchst 33258. Fluorescent signals were observed with a Leica DMI 6000B microscope. The specificity of α-tubulin immunolabelling was controlled by omitting the primary antibody.

### Mouse *Adgrl2* inactivation

*Adgrl2*^*+/−*^ mice were purchased from INFRAFRONTIER (Neuherberg, Germany). *Adgrl2* was inactivated by insertion of a LacZ-neo cassette in the DNA sequence (strain B6;129P2-Adgrl2^tm1Dgen^/H, Mary Lyon Centre, MRC Harwell, Oxfordshire, UK). Mice were maintained at the animal facility of the Rouen Medical University. All rodent work was performed with the consent of the Animal Ethics Committee, Rouen Faculty of Medicine. All animals were fed a standard diet and maintained in a pathogen-free environment on a 12 h light/12 h dark cycle.

For genotyping, at least 3 mm of the mouse tail was cut, genomic DNA was then extracted using the NucleoSpin® Tissue kit (Macherey-Nagel, Hœrdt, France) and quantified using a NanoVue TM spectrophotometer (GE Healthcare Life Sciences, Pittsburgh, PA, USA). To make sure that mice were accurately genotyped, three genotyping assays were developed, the first multiplex PCR being designed to simultaneously detect the wild-type and targeted allele, the second being designed to detect the targeted allele only and the third to amplify the wild-type allele only. PCR primers and conditions are available upon request.

### Magnetic resonance imaging (MRI)

To evaluate long-term brain lesions, MRI analyses were carried out on female and male adult (P45) mice using a Pharmascan 7 T (Bruker, Wissenbourg, France). T2-turboRARE 3D weighted images were acquired using a multislice multiecho sequence: TE/TR 31.5 ms/1500 ms. Quantification of morphometric brain characteristics was done using ImageJ (NIH software v1.45r, National Institute of Health, Bethesda, MD, USA) previously upgraded with the Bruker plugins. Image analyses provided access to qualitative and quantitative neuroanatomical criteria including cerebrum width, cerebrum volume, lateral ventricle volume and mid-sagittal anteroposterior vermis diameter.

### Statistical analyses

Statistical analyses were performed using the biostatistics Prism software (GraphPad Inc.). Tests used for each experiment, the number of independent experiments and *p*-values are detailed in Additional file 3: Table S3.

## Results

### Neuropathological examination reveals extreme microcephaly and RES

**Fig. 1:**
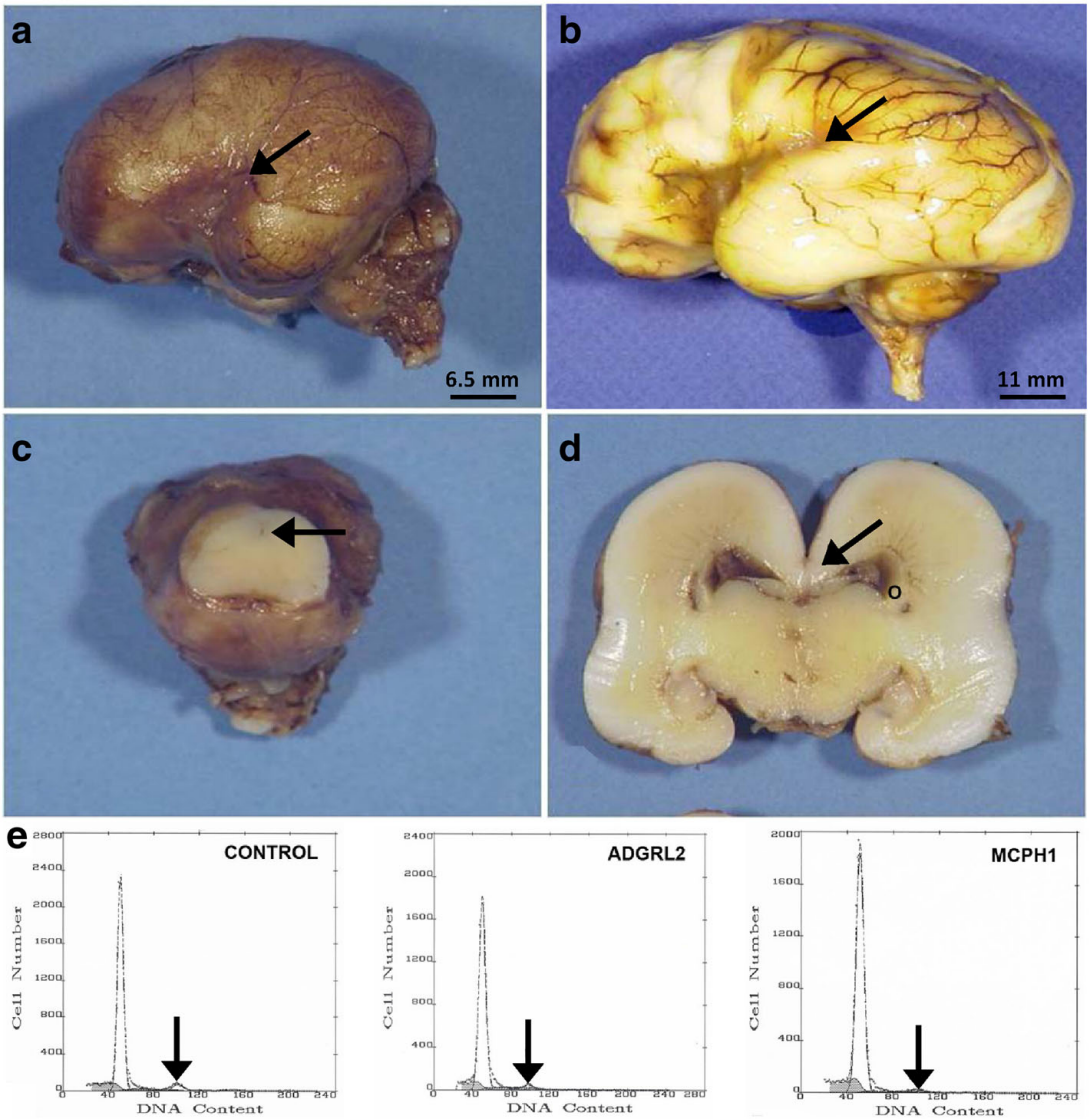
Main macroscopic findings. **a** Left side of the brain showing lissencephaly with no gradient of severity, with a short Sylvian fissure reduced to a dimple (black arrow). **b** Comparison with an age-matched control brain aged 26WG, where all primary fissures are present, with a posteriorly closed Sylvian fissure (black arrow). **c** Macroscopic view of the mesencephalon, displaying a punctiform aqueduct of Sylvius (black arrow) beneath the cerebellum, which forms a single non-foliated mass corresponding to an RES. **d** On a coronal section passing through the cerebral hemispheres at the level of the hippocampi, the third ventricle is severely narrowed, the corpus callosum is present (black arrow) and the hippocampi appear hypoplastic. The circle represents the LGE. **e** Flow cytometry cell cycle profiles in the LGE at 26WG. Left: control fœtus, middle: *ADGRL2* mutated fœtus and right: *MCPH1* mutated fœtus. Arrows indicate G2/M phases

**Fig. 2:**
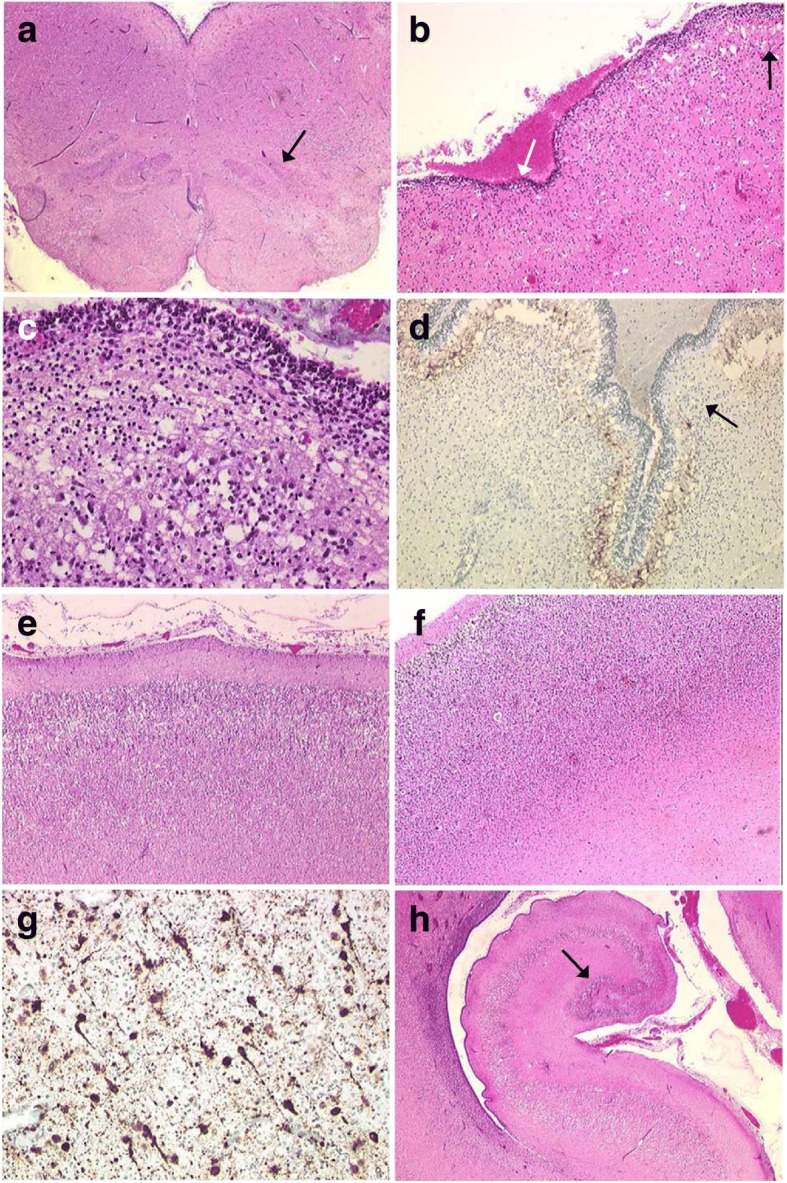
Histological hallmarks of brain lesions. **a** Histological section passing through the medulla, where the olivary nuclei are poorly convoluted and hypoplastic (arrow) [H&E, OM × 15]. **b** In the most severely affected areas, the cerebellar cortex is rudimentary, with a strongly hypoplastic transient external granular cell layer (white arrow) [H&E, OM × 100]. **c** With higher magnification, almost no discernible Purkinje cell and internal granular cell layers [H&E, OM × 200]. **d** Focally missing Purkinje cells in the less affected areas (black arrow) [anti-calbindin immunolabelling, OM × 100]. **e** Thin six-layered cortical plate [H&E, OM × 25]. **f** Comparison with the control brain, where the cortical plate is thicker, with a higher density of neurons [H&E, OM × 25]. **g** Anti-MAP2 immunohistochemistry revealing numerous persistent migrating neurons in the intermediate zone [OM × 400]. **h** Hypoplastic hippocampi, the dentate gyrus being reduced to a small mass of granular neurons (arrow) [H&E, OM × 25]. H&E: haematoxylin-eosin staining. OM: original magnification

A 26-year-old woman, gravida II, para I, underwent routine ultrasonography at 22WG which displayed intra-uterine growth retardation, polyhydramnios, decreased fœtal movements, and microcephaly with gyral abnormalities and pontocerebellar hypoplasia. An MRI carried out at 25WG confirmed these abnormalities, and a medical termination of the pregnancy was achieved at 26WG in accordance with the French law and after approval by our local ethical committee. Array CGH using an Agilent array 105 K performed on amniotic fluid cells revealed a normal female karyotype, 46,XX. Both unrelated parents had no personal or familial medical history. General autopsy confirmed growth failure (<5th percentile) with malposition of the extremities but with no visceral anomalies [24]. Brain weight was ≪ 3rd centile (28.5 g, normal weight = 146.21 g +/− 21.69) with an infratentorial weight and a transverse cerebellar corresponding to 13WG [23, 34]. The brain surface was agyric. Sylvian fissures were short, dimple-shaped and vertically oriented (Fig. 1a and b). Olfactory tracts and optic chiasm were present. At the level of the cerebral peduncles, the aqueduct of Sylvius was identified and the cerebellum was replaced by a single non-foliated mass corresponding to RES (Fig. 1c), the vermis being indiscernible and the cerebellar hemispheres entirely fused across the midline. On coronal sections, the corpus callosum was identified, but the hippocampi were hypoplastic (Fig. 1d). Histological examination of the spinal cord revealed hypoplasia of the corticospinal tracts. In the mesencephalon, pons and medulla, neuronal density was diminished within the cranial nerve nuclei. Olivary nuclei were poorly convoluted and hypoplastic, but with no olivary ectopias (Fig. 2a). In the cerebellum, the dentate nuclei were non-convoluted but not fused. The cerebellar cortex was rudimentary, the transient external granular cell layer being particularly thin (Fig. 2b). Neither Purkinje cell nor internal granular cell layers were clearly identified (Fig. 2c) and calbindin immunohistochemistry revealed scattered Purkinje cells irregularly distributed throughout the cerebellar cortex (Fig. 2d). In the cerebral hemispheres, neuronal density was decreased in the cortical plate, particularly in layer II but with normal cortical lamination (Fig. 2e and f). Layer I was cellular, with abnormal persistence of the transient external granular cell layer and scarce reelin-positive Cajal-Retzius cells compared to the control case. The calretinin antibody also immunolabelled Cajal-Retzius cells, along with dispersed positive neurons distributed throughout the telencephalon. Layers IV, V and VI were made of immature neurons. Vimentin-positive radial glial fibres persisted in the intermediate zone, which contained numerous migrating MAP2-positive neurons and GFAP-positive astrocytes, with no axonal spheroids (Fig. 2g). The hippocampal uncus was also abnormal, the dentate gyrus being short and thick, but with a preserved pyramidal cell layer (Fig. 2h). No lesion was observed in the basal ganglia and thalami. Thus, histological lesions were suggestive of a defect in neural cell production along with abnormalities of radial and tangential migration. Proliferative indices (PI) evaluated by means of flow cytometry from LGE in normal control, *ADGRL2* mutated and *MCPH1* mutated fœtuses at the same term revealed that in the control brain, LGE cell PI was 9.6%, with 4.9% of cells in phase S. In the *ADGRL2* mutated patient, PI was measured at 9.1% that did not differ from the normal control, but with a slightly higher percentage of S-phase cells evaluated at 7%. Conversely, PI in the *MCPH1* mutated brain was severely decreased by a factor of 2, calculated at 5.1%, with a reduced S phase measured at 3.8% (Fig. 1e). These results clearly indicate that proliferative capacities to provide post-mitotic neuroblasts are not affected in the *ADGRL2* mutated fœtus.

### Whole exome sequencing reveals a de novo variation in the *ADGRL2* gene

**Fig. 3:**
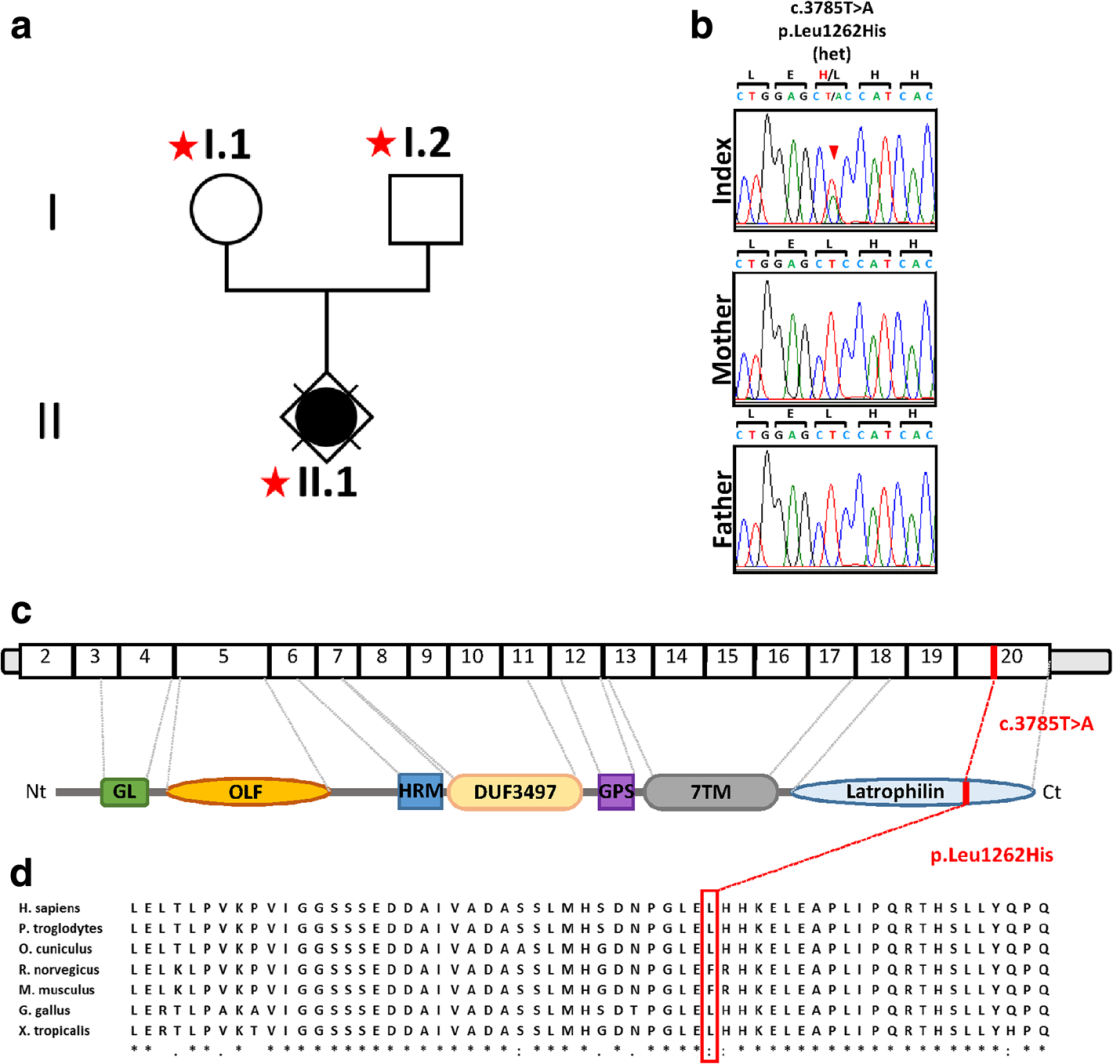
Identification of a de novo heterozygous variant in *ADGRL2*. **a** Pedigree structure of the family. Red stars depict individuals subjected to WES. **b** WES identified a *ADGRL2* c.3785T>A heterozygous variant resulting in a p.(Leu1262His) amino acid substitution, confirmed de novo by Sanger sequencing of proband and parents. **c** Schematic representation of ADGRL2 mRNA and protein. ADGRL2 contains a galactose binding lectin domain (GL), an olfactomedin-like domain (OLF), a domain present in hormone receptors (HRM), a domain of unknown function (DUF), a G-protein coupled receptor proteolytic site domain (GPS), 7 transmembrane domains (TM) and a cytoplasmic latrophilin domain. The variant (red) was localized in the exon 20, the resulting amino acid substitution occurred in the latrophilin domain. **d** Phylogenic conservation of the C-terminal domain. The position of the amino acid substitution is indicated by the red rectangle. Nt: amino-terminal; Ct: carboxy-terminal

In the absence of clinical symptoms in the parents and because no genetic cause is known for this brain malformation association, a germline de novo variation was likely to be responsible for this very unusual neuropathological phenotype. To identify such variations, a comparative WES analysis of the fœtus and her healthy parents was performed (Fig. 3a). Parental links were verified by microsatellite analysis. Across the three exomes, an average of 5.6Gb with 97% of mappable sequences was obtained, a mean read depth of 61×, 89% of bases were covered to a minimum depth of 10 and 89% of the read bases had a Qscore above 30 (Additional file 1: Table S1). More than 65% of reads were On-target captured. On average, 17,500 exonic variants were identified per exome. First, variants with a read depth of less than 10× and a Qscore below 30 were filtered out. The resulting variants were filtered according to their null frequency in the general population from 1000 Genomes Project, Exome Aggregation Consortium (ExAC 0.3.1) and genome Aggregation Database (gnomAD r2.0.2). When the variants detected in the parents were subtracted from those encountered in the patient to identified de novo variations, only one variation remained: c.3785T>A;p.(Leu1262His) in exon 20 of the *ADGRL2* (NM_012302.4, MIM#607018). The missense variation was predicted to be damaging by MutationTaster, SIFT, and PROVEAN, suggestive of its pathogenicity. According to the ExAC consortium, *ADGRL2* is loss-of-function intolerant (pLI = 1). No other variant was found when analysing trio data under recessive and X-linked hypotheses. This heterozygous variant was then validated by Sanger sequencing in the patient and its absence in the peripheral blood DNA of the parents indicating that the c.3785T>A variation occurred de novo (Fig. 3b). *ADGRL2* (adhesion G protein-coupled receptor L2) gene previously called *LPHN2* encodes the latrophilin 2 protein [26] and maps to chromosome 1, at 1p31.1. It belongs to the adhesion class G protein-coupled receptor (GPCR) family. The three human ADGRLs (1, 2 and 3) have previously been identified as the functional receptors of α-latrotoxin, the major neurotoxin of the black widow spider venom [64, 65, 69]. ADGRLs are evolutionary conserved across species and share similar protein architecture characterized by three major domains: a long glycosylated N-terminal extracellular domain, seven Trans Membrane Regions (TMRs), and a long cytoplasmic tail (Fig. 3c) [39, 42]. The de novo variation identified by comparative WES is located in the intracellular conserved domain (Fig. 3d).

### *ADGRL2* resequencing in a panel of 29 unrelated RES-affected individuals fails to detect any pathogenic variant

As RES was one of the main lesions observed in the fœtus, the hypothesis that *ADGRL2* variants could be responsible for a wider phenotypic spectrum was raised. All coding exons of the *ADGRL2* gene by means of the Sanger technique were sequenced in 29 unrelated fœtuses affected with RES alone or with associated mesencephalosynapsis (atresia-forking of the aqueduct of Sylvius and fusion of the colliculi), diencephalosynapsis (atresia of the 3rd ventricle with collapse of the thalami), holoprosencephaly or encephalocele [56]. No variant was detected in these 29 fœtuses.

### *Adgrl2* is early expressed during chicken and mouse development

**Fig. 4:**
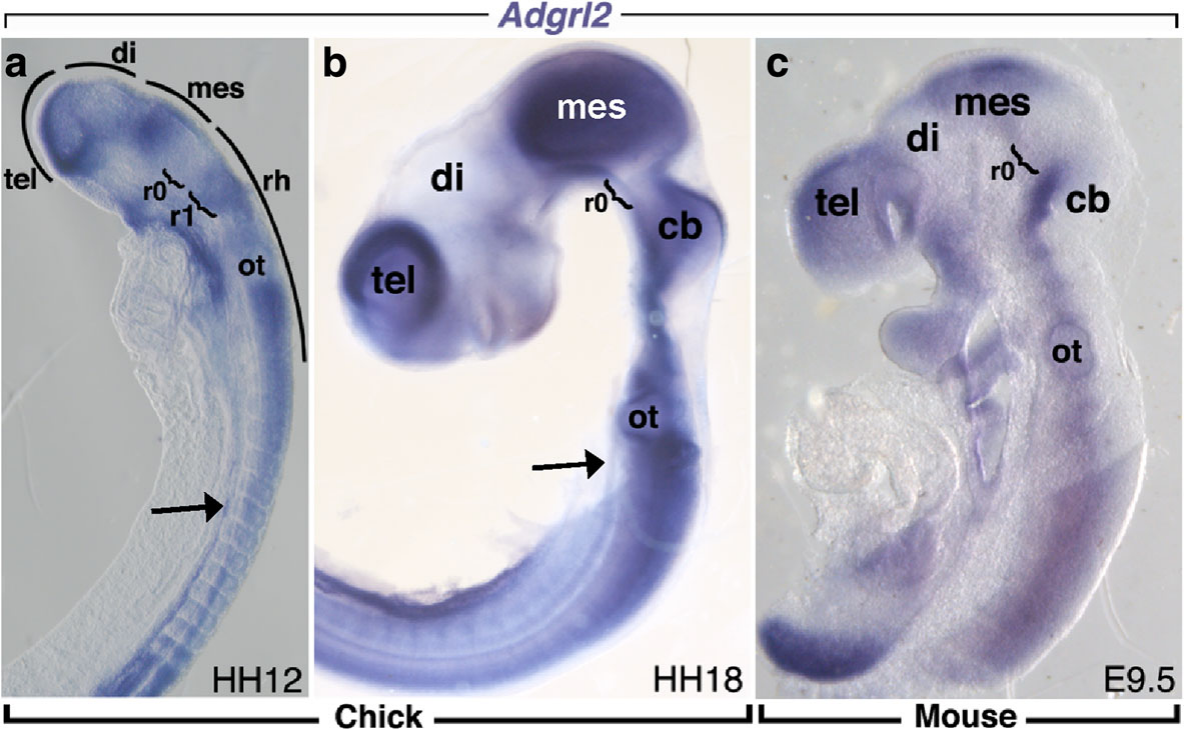
Expression of the *Adgrl2* gene during early development in chicken and mouse embryos. **a**, **b** Spatiotemporal expression of *Adgrl2* on a HH12 whole chick embryo (**a**) and on an HH18 chick dissected neural tube (**b**). At HH12, strong expression is seen throughout the neural tube while weak expression is observed in the diencephalon and isthmocerebellar region (black bracket). At HH18, strong expression is still present in the telencephalon, mesencephalon and cerebellum. Very low expression is still observed in the diencephalon and isthmocerebellar region (black bracket). **c** Similar strong expression of *Adgrl2* is observed in the telencephalon, mesencephalon and cerebellum of a 9.5 mouse embryo. di: diencephalon; cb: cerebellum; mes: mesencephalon; ot: otic vesicle; r0: isthmic organizer region; r1: rhombomere 1; rh: rhombencephalon; tel.: telencephalon

*Aldgrl2* expression was investigated in chicken and mouse embryos just after brain segmentation has taken place (HH12-HH18 for chicken embryo and E9.5 for mouse embryo). In the HH12 chick embryo, *Adgrl2* was expressed along the neural tube with an intense expression in the telencephalic vesicles (both in the future cerebral mantle and in the germinal zones), in the mesencephalon and in the rhombencephalon. Low levels of in situ hybridization signals for *Adgrl2* were detected in the diencephalic vesicle and in the isthmic organizer region (r0), at the mesencephalon-metencephalon boundary (Fig. 4a). Significant expression was also observed along the notochord (Fig. 4a, b), with increased expression at subsequent developmental stages. At HH18, on dissected brains, strong *Adgrl2* expression persisted in the telencephalon, mesencephalon and in the developing cerebellum, but remained weak in the diencephalon and at the level of isthmic organizer region (Fig. 4b). In the developing cerebellum, *Adgrl2* expression was observed in the rhombic lips and transient external granular cell layer.

Similar *Adgrl2* expression domains were observed in mouse embryos at E9.5. Mouse *Adgrl2* was strongly expressed in the telencephalon, mesencephalon and cerebellum, but absent in the diencephalon and at the mesencephalon-metencephalon boundary (Fig. 4c). These first expression studies of *Adgrl2* reveal that mouse and chicken *Adgrl2* display similar region-specific expression in the cephalic vesicles and that *Adgrl2* is involved in a highly conserved mechanism that is crucial during early development of the telencephalon and cerebellum.

### *ADGRL2* is expressed from early human development

In human embryos, at 6^th^, 9^th^ and 10^th^ PCW, strong immunoreactivity was observed in almost all organs and tissues, notably in the liver parenchyma, heart, primary bronchi, digestive epithelium, nephrogenic blastema, smooth and striated muscle cells, vascular endothelium, as well as in mesenchymal tissues, especially the cartilaginous cells of the head, neck, thorax and of the axial skeleton. ADGRL2 immunoreactivity was strong in the seminiferous cords of the testes and in the epithelium of the epididymis from the 6^th^ PCW, and from the 10^th^ PCW in ovary germ cells. From 14WG onward, oogonia and follicular cells were intensively immunoreactive along with the ovarian superficial epithelium. From 18WG to birth, diffuse immunolabelling persisted in the primordial follicles (oogonia and follicular cells, Additional file 4: Figure S1a, b), and in the Leydig cells of the ovarian hilum (Additional file 4: Figure S1c). In male fœtuses, spermatogonia, Sertoli and Leydig cells, as well as interstitial mesenchymal testicular matrix, were strongly immunolabelled from 18WG to birth (Additional file 4: Figure S1d).

**Fig. 5:**
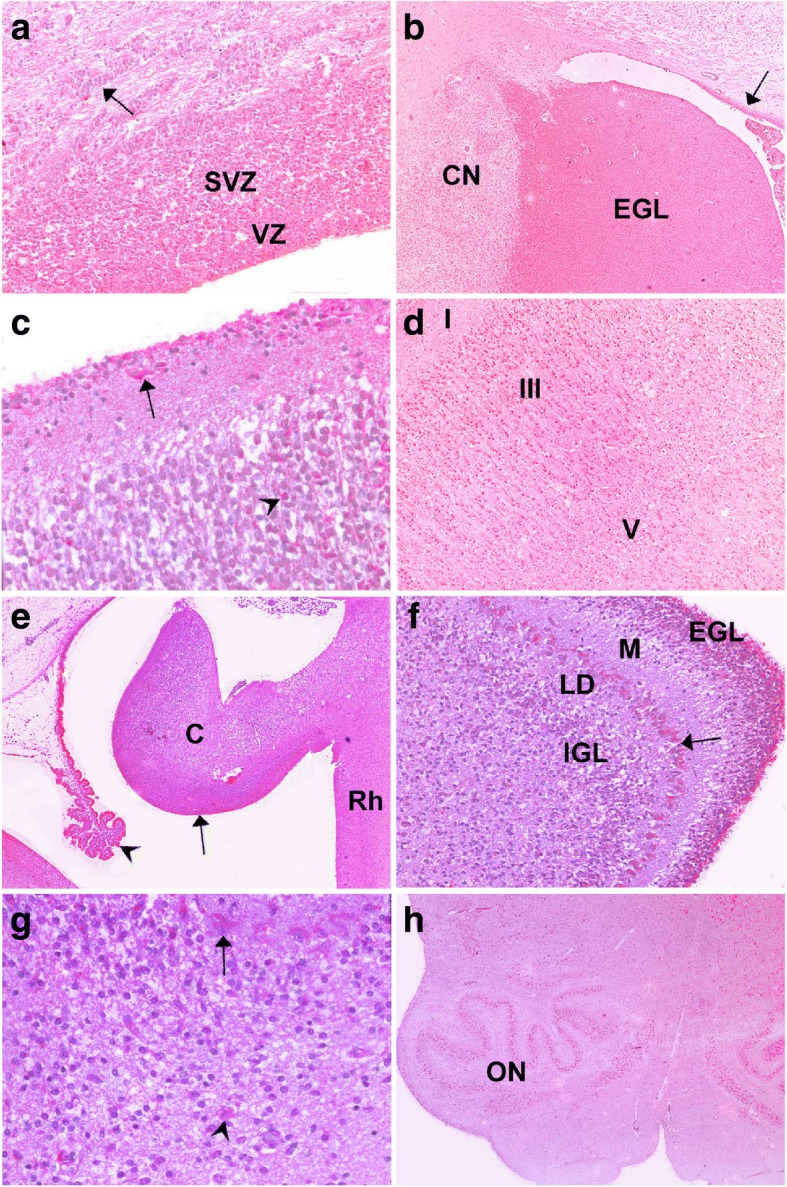
ADGRL2 immunohistochemistry in the normal developing human brain. **a** Strong immunoreactivity of stem cells in the VZ and of intermediate progenitors in the SVZ, as well as in several migrating neurons (arrow) [OM × 100]. **b** Immunopositivity of LGE at 18WG [OM × 25]. **c** 18WG onward positive Cajal-Retzius cells in layer I (thick arrow) and cortical neurones (arrow head) [OM × 250]. **d** Immunoreactive differentiating pyramidal neurons of the layers III and V [OM × 25]. **e** Intense immunolabelling of the developing cerebellum at 6PCW, predominating in the ventricular zone (thick arrow) and in the choroid plexuses (arrow head) [OM × 25]. **f** At 24WG, strong immunoreactivity of the transient external granular cell layer and in Purkinje cells (arrow), but with no positivity in the developing internal granular cell layer [OM × 250]. **g** Only positive Golgi II neurons at 32WG in the internal granular cell layer (arrow head), Purkinje cells remaining strongly immunoreactive (thick arrow) [OM × 400]. **h** Diffuse neuronal immunoreactivity in the olivary nuclei from 25WG [OM × 25]. OM: original magnification; VZ: ventricular zone; SVZ: subventricular zone; CN: caudate nucleus; LGE: lateral ganglionic eminences; C: cerebellum; Rh: rhombencephalon; EGL: external granular cell layer of the cerebellar cortex; M: molecular layer; LD: lamina dissecans; IGL: internal granular cell layer of the cerebellar cortex, ON: olivary nucleus

Apart from gonad immunohistochemistry, immunohistochemical analyses were restricted to brain anatomical structures from 13WG onwards. In the cerebral hemispheres, the neuroepithelium was intensively immunoreactive from the 6^th^ PCW to 24WG, with a progressive increase in cell immunoreactivity in the subventricular zone (Fig. 5a). LGE were moderately positive from 13WG, became intensely immunolabelled until 24WG (Fig. 5b) and became negative by around 30WG, whereas ependymal cell lining was positive from 30 to 34WG. In the cortical plate, the tangential fibre network of layer I was positive as early as 6PCW, with few positive neurons in the developing cortical plate. From 16WG, Cajal-Retzius cells were positive (Fig. 5c), and from 22WG to 34WG pyramidal neurons differentiated from layers III and V (Fig. 5d), whereas ADGRL2 immunoreactivity remained weak in layers II and IV until birth. Radial glia was immunolabelled from 13WG to 30WG. Positive-ADGRL2 migrating neurons were observed in the intermediate zone until 24WG, a developmental stage corresponding to radial migration termination. At birth, a few migrating interneurons were still observed. ADGRL2-positive neurons were scattered throughout the basal ganglia, thalami and hypothalamic nuclei. At the infratentorial level, transiently immunoreactive fibres and neurons were observed within and around the colliculi, and in the substantia nigra between 13 and 30WG. A transient ADGRL2 immunoreactivity was also present in the pontine transverse fibres and pontine nuclei from 13WG to 22WG. In the cerebellum, the dorsal neuroepithelium of the 4th ventricle and the rhombic lips were strongly immunolabelled from the 6th PCW to 16WG (Fig. 5e), then gradually disappeared by 20WG. Bergman glia, migrating Purkinje and cerebellar deep nuclei positive neurons were apparent from 10 PCW. Purkinje cells were positive until birth (Fig. 5f). From 13WG onward, the transient external granular cell layer was strongly immunolabelled, and became negative at 34WG. In the internal granular cell layer, only Golgi II neurons were positive (Fig. 5g). Cranial nerve nuclei of the pons and medulla became positive from the 24th WG, as well as olivary nuclei, whose immunoreactivity persisted until birth (Fig. 5h). To summarize, ADGRL2 immunoreactivity was mainly observed in the germinal zones both at the supra- and infratentorial levels, and to a lesser degree, in some discrete migrating neuron populations and more mature anatomical structures.

### *ADGRL2* expression is normal in patient amniocytes carrying the c.3785T>A variant

ADGRL2 is post-transcriptionally processed, the full length precursor being autoproteolytically cleaved in two independent fragments (N-terminal fragment, 70-75 kDa and C-terminal fragment, 105-110 kDa) which are addressed to the plasma membrane [72]. To investigate whether the missense variant identified by WES could alter ADGRL2 protein stability or processing, protein expression was evaluated by western blot experiments on the patient amniocytes collected at the time of amniocentesis performed at 21WG, by comparison with amniocyte ADGRL2 expression from control fœtuses at the same developmental stage. As shown in Additional file 5: Figure S2, no differences in ADGRL2 expression and cleavage were observed between the patient’s and the control amniocytes, indicating that the *ADGRL2 * c.3785T>A variant does not alter ADGRL2 expression or processing, but rather impairs its functionality.

### Intracellular Ca^2+^ release in response to ligand activation is altered in patient *ADGRL2* amniocytes

**Fig. 6:**
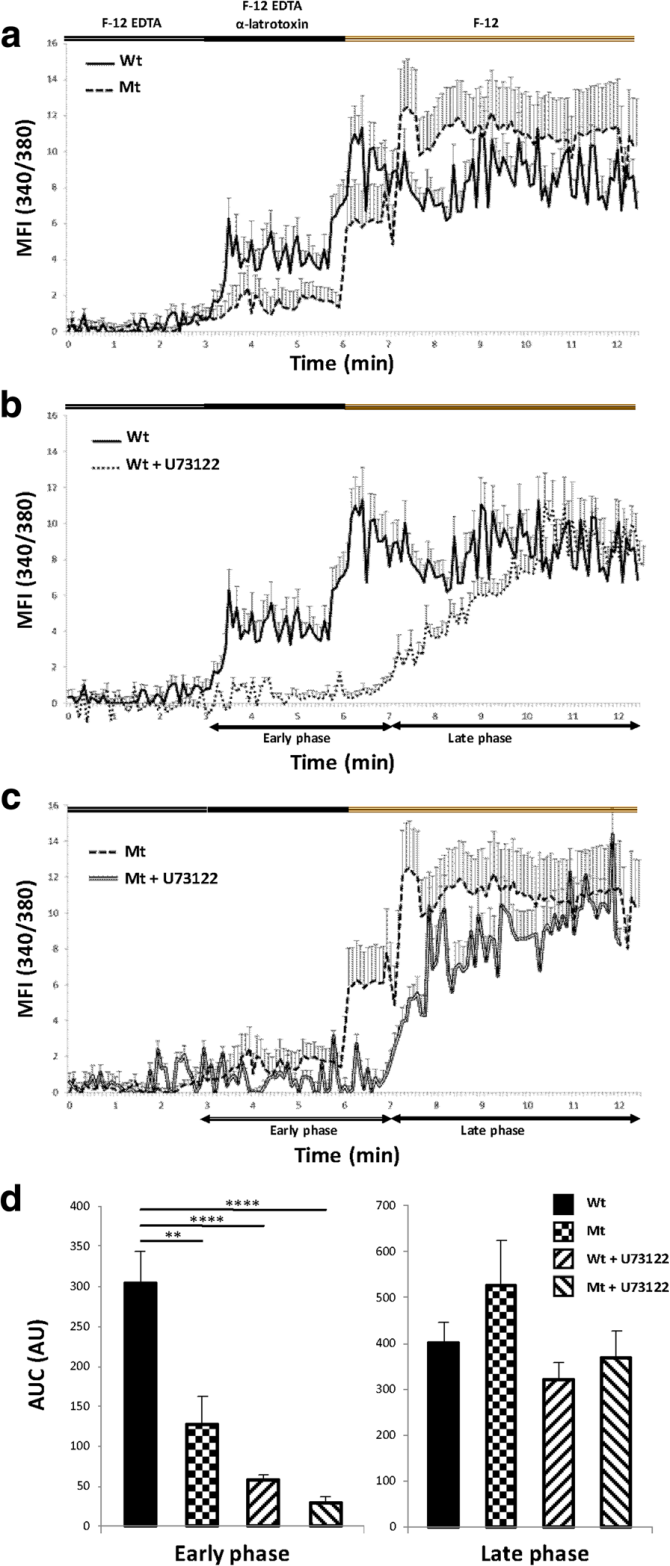
Signal transduction coupled to G protein is altered in mutant *ADGRL2* amniocytes. Intracellular calcium levels were monitored by microfluorimetry using the ratiometric Fura-2 AM calcium probe and results expressed as mean fluorescence intensity (MFI). **a** α-latrotoxin (1 nM) was applied to wild-type (Wt) and mutant (Mt) cultured amniocytes under extracellular chelated-calcium conditions (EDTA 4 mM). Three minutes after α-latrotoxin administration, cells were perfused without EDTA to restore extracellular calcium levels. **b** α-latrotoxin (1 nM) was applied to Wt amniocytes under chelated-calcium conditions (EDTA 4 mM). Cultured cells were pre-incubated or not with the phospholipase C inhibitor U73122 (10 μM). Three minutes after α-latrotoxin administration, cells were perfused without EDTA to restore extracellular calcium levels. **c** α-latrotoxin (1 nM) was applied to Mt amniocytes under chelated-calcium conditions (EDTA 4 mM). Cultured cells were pre-incubated or not with the phospholipase C inhibitor U73122 (10 μM). Three minutes after α-latrotoxin administration, cells were perfused without EDTA to restore extracellular calcium levels. **d** Quantification and statistical analysis of intracellular calcium levels from the early and late phases in response to α-latrotoxin stimulation. Areas under the curves (AUC) were expressed in arbitrary units (AU). Each value represents the mean (±S.E.M.) of 30 cells. *, *p* < 0.05; **, *p* < 0.01, vs Wt amniocytes using one-way ANOVA test

The exogenous ligand α-latrotoxin induces Ca^2+^_i_ elevation by two mechanisms, which are not mutually exclusive. The first is ADGRL2-receptor specific: the subsequent receptor transduction pathway involves a G protein coupled to activation of phospholipase C (PLC), production of inositol-triphosphate (IP3) and release of Ca^2+^ from intracellular stores [4, 42]. The second is a consequence of the ionophoric properties of α-latrotoxin [3, 30]. Toxin tetramers forming transmembrane pores induce passive extracellular Ca^2+^ (Ca^2+^_e_) influx into the cell. Moreover, α-latrotoxin binds to at least two types of receptors associated with Ca^2+^_i_ release. Neurexins are exclusively activated by α-latrotoxin in a Ca^2+^_e_ dependent manner [17, 38], whereas ADGRLs also bind the toxin in the absence of Ca^2+^_e_ [4, 31]. To specifically study ADGRLs, media complemented with 4 nM EDTA (F12-EDTA condition) to induce Ca^2+^_e_ chelation were used [3]. To study the functionality of the ADGRL2 receptor, an assay was carried out using microfluorimetry that reflected mutated receptor ability to transduce Ca^2+^_i_ [66]. Alpha-latrotoxin was used as a ligand on fura-2 loaded amniocytes obtained from the fœtus carrying the *ADGRL2* variation and wild-type amniocytes from control fœtuses at the same term (Fig. 6). To discriminate between intra- and extra-cellular pools of calcium [3], amniocytes were cultured in F12 media in absence or presence of 4 mM EDTA in F12-EDTA condition. Application of 1 nM α-latrotoxin on control cells (Wt) induced a cytosolic Ca^2+^_i_ increase within the first 30 s (Fig. 6a; full line). Three minutes after 1 nM α-latrotoxin exposure, EDTA removal from the perfusion medium was associated with another cytosolic Ca^2+^_i_ increase, which reached a maximum stable value (Fig. 6a). The effect of 1 nM α-latrotoxin on cytosolic Ca^2+^_i_ under F12-EDTA condition was more than two times lower in mutant cells (Mt) (Fig. 6a, d; dotted line). As found in Wt cells, a similar second phase of cytosolic Ca^2+^_i_ increase was observed after removal of EDTA (Fig. 6a, d).

To investigate in greater details the Ca^2+^_i_ mobilization associated with the ADGRL2 receptor, Wt and Mt. cells were co-incubated in the presence of the PLC inhibitor, U73122 (Fig. 6b, c) [10]. In F12-EDTA condition, 10 μM U73122 abrogated the cytosolic Ca^2+^_i_ increase induced by α-latrotoxin in both Wt and Mt. cells (Fig. 6b, c). In contrast, U73122 had no significant impact on the late phase of Ca^2+^_i_ increase resulting from EDTA removal (Fig. 6d).

### *ADGRL2* c.3785T>A variant is responsible for signal transduction alteration

To demonstrate the deleterious effect of the variant independently from the patient genetic background, HeLa cells were used as an exogenous cellular model in which the candidate gene was overexpressed, due to the very low expression of ADGRL2 in these cells. As human ADGRL2 cDNA is toxic to bacteria, cloning was not possible and a previously described construct derived from rat *Adgrl2* homolog, pcDCIRL-2 coupled to GFP, was used [31]. To generate mutant Adgrl2 clones, a histidine was introduced at position 1262 using site-directed mutagenesis. First, the correct processing and trafficking of the GFP coupled receptor to the plasma membrane of transfected HeLa cells was verified by confocal microscopy and transfection efficiency by western blot (Additional file 6: Figure S3a, b). However, in F-12 EDTA condition, microfluorimetry experiments performed with pcDCIRL-2-GFP cells did not display any cytosolic Ca^2+^_i_ increase after α-latrotoxin exposure: only a late phase of Ca^2+^_i_ increase was observed. Because C-terminal GFP could induce steric hindrance and inhibit Adgrl2 signal transduction coupled to G protein in response to ligand binding, the wild-type pcDCIRL-2 construct lacking the GFP sequence was transfected into HeLa cells. The resulting cytosolic Ca^2+^_i_ profiles after α-latrotoxin exposure were very similar to those obtained with Wt amniocytes (Fig. 6a,). In contrast, HeLa cells overexpressing mutant Adgrl2 presented exclusively the late-phase Ca^2+^_i_ increase (Additional file 6: Figure S3c), confirming that *ADGRL2* c.3785T>A variant is responsible for the alteration of the signal transduction coupled to G-protein in response to ligand binding.

### *Adgrl2*^−/−^ mice die at E15.5

An *Adgrl2* knock-out (KO) mouse model was used to outline the phenotype associated to *ADGRL2* inactivation. Adult heterozygous mice were fertile and survived for more than 1 year, but had a shorter fertility lifespan compared with wild-type mice, a few months vs 2 years, respectively. *Adgrl2*^*+/***−**^ F2 mice were mated in order to obtain *Adgrl2*^**−**/**−**^. A balanced sex ratio distribution was observed in the 15 litters analysed. Nevertheless, *Adgrl2* genotyping of mouse pups was not in agreement with the Hardy–Weinberg equilibrium, confirming embryo lethality of *Adgrl2*^**−**/**−**^ mice as previously described by the provider, who suggested an embryonic lethality at ~E15.5 (χ^2^ = 49.35). In contrast, E15 embryos *Adgrl2* genotyping was concordant with the Hardy–Weinberg equilibrium (χ^2^ = 1.81), even if a slight deviation began to occur.

### MRI findings in *Adgrl2*^+/−^ adult mice confirm microcephaly, hypoplasia of the vermis and mesencephalon

**Fig. 7:**
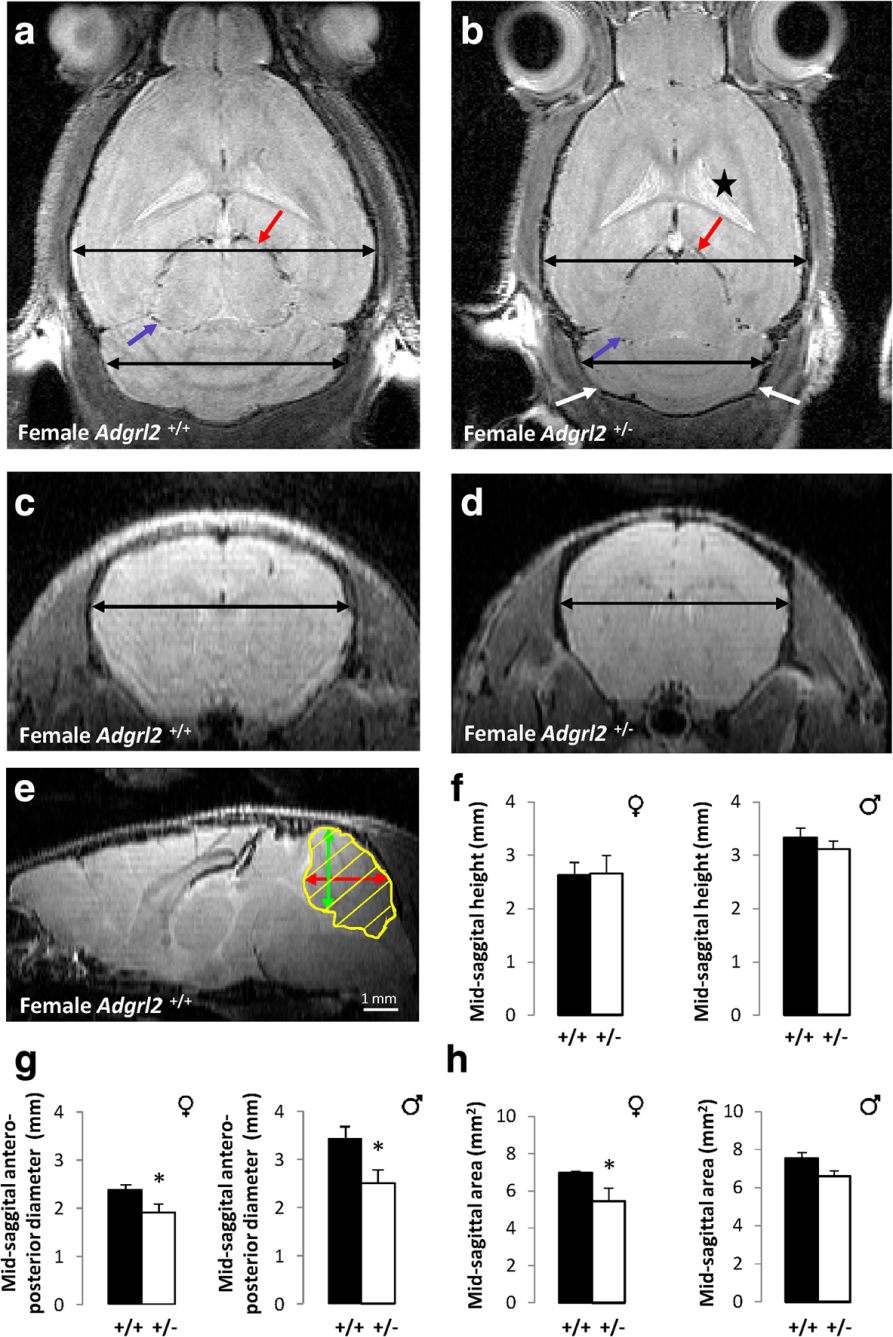
Main representative images and quantitative findings obtained from MRI performed in *Adgrl2*^*+/+*^ and *Adgrl2*^*+/−*^ male and female adult mouse brains. **a-b** Horizontal T2-weighted images passing through the mesencephalon of *Adgrl2*^*+/+*^ mice. **a** revealing in *Adgrl2*^*+/−*^ mice **b** a defect in tectum growth with insufficiently developed colliculi (red arrow) and pes pedunculi (blue arrow) with a growth defect of the telencephalon (line corresponding to bi-parietal diameter) responsible for *a vacuo* ventricular dilatation (asterisk), and of the cerebellum (line corresponding to transverse diameter) with smaller cerebellar hemispheres (white arrows). **c** T2-weighted images passing through the optic chiasm in *Adgrl2*^*+/+*^ *mice.* **d** Deficient frontal growth in *Adgrl2*^*+/−*^ mice (thick line). **e** Schematic representation of the different measurements performed on *Adgrl2*^*+/+*^ and *Adgrl2*^*+/−*^ male and female adult mouse vermis (height in green, anterior-posterior diameter in red, and area in yellow) revealing no difference in heights in *Adgrl2*^*+/+*^ and *Adgrl2*^*+/−*^ females compared to males (**f**), whereas significant differences in anterior-posterior diameters were apparent both in females and males (**g**), but with significant differences in vermis areas between *Adgrl2*^*+/+*^ and *Adgrl2*^*+/−*^ female mice only contrary to *Adgrl2*^*+/+*^ and *Adgrl2*^*+/−*^ males (**h**)

Comparative analyses of horizontal T2-weighted images passing through the mesencephalon at the level of red nuclei revealed a defective growth of the tectum (consisting of less developed superior colliculi) and of the tegmentum (with narrowed cerebral peduncles) in *Adgrl2*^*+/***−**^ female adult mice compared to wild type female mice (Fig. 7a, b). A growth failure of the cerebellum was also observed, with a smaller cerebellar transverse diameter (Fig. 7a, b). On transversal planes passing though the widest cerebellar transverse diameter, para-flocculonodular lobes were visualized in *Adgrl2*^*+/***−**^ animals, reflecting a defect in anterior-posterior growth of the cerebellum. At the supratentorial level, a mild dilatation of the lateral ventricles due to insufficient expansion of the brain parenchyma (as reflected by a diminished bi-parietal diameter) was also noted, arguing for microcephaly. Quantitative measurements of cerebral volumes confirmed a significant reduction in brain volumes (wild-type mice: 392 ± 8 mm^3^ vs *Adgrl2*^*+/***−**^: 332 ± 7 mm^3^; *p* < 0.001; Additional file 3: Table S3). Microcephaly extending to the parietal lobes was also present in males, with a moderate enlargement of the subarachnoid spaces in the anterior regions, but with no shape anomalies of the lateral ventricles. Examination of T2-weighted transversal planes passing through the optic chiasm revealed a defect in superior and middle frontal gyrus expansion, as well as abnormally-shaped lateral ventricles in female *Adgrl2*^*+/***−**^ adult mice (Fig. 7c, d). Quantitative analyses performed on vermis median sagittal planes (heights, anterior-posterior diameters and global vermis areas, Fig. 7e) revealed that, contrary to heights that did not significantly differ between wild-type and heterozygous males or females (Fig. 7f), anterior-posterior diameters were significantly reduced when comparing wild-type and heterozygous males and wild-type and heterozygous females (Fig. 7g). Significantly reduced vermis areas were observed in female *Adgrl2*^*+/***−**^ adult mice (Fig. 7h).

### *ADGRL2* c.3785T>A variant increases cell adhesion properties and reduces cell motility

**Fig. 8:**
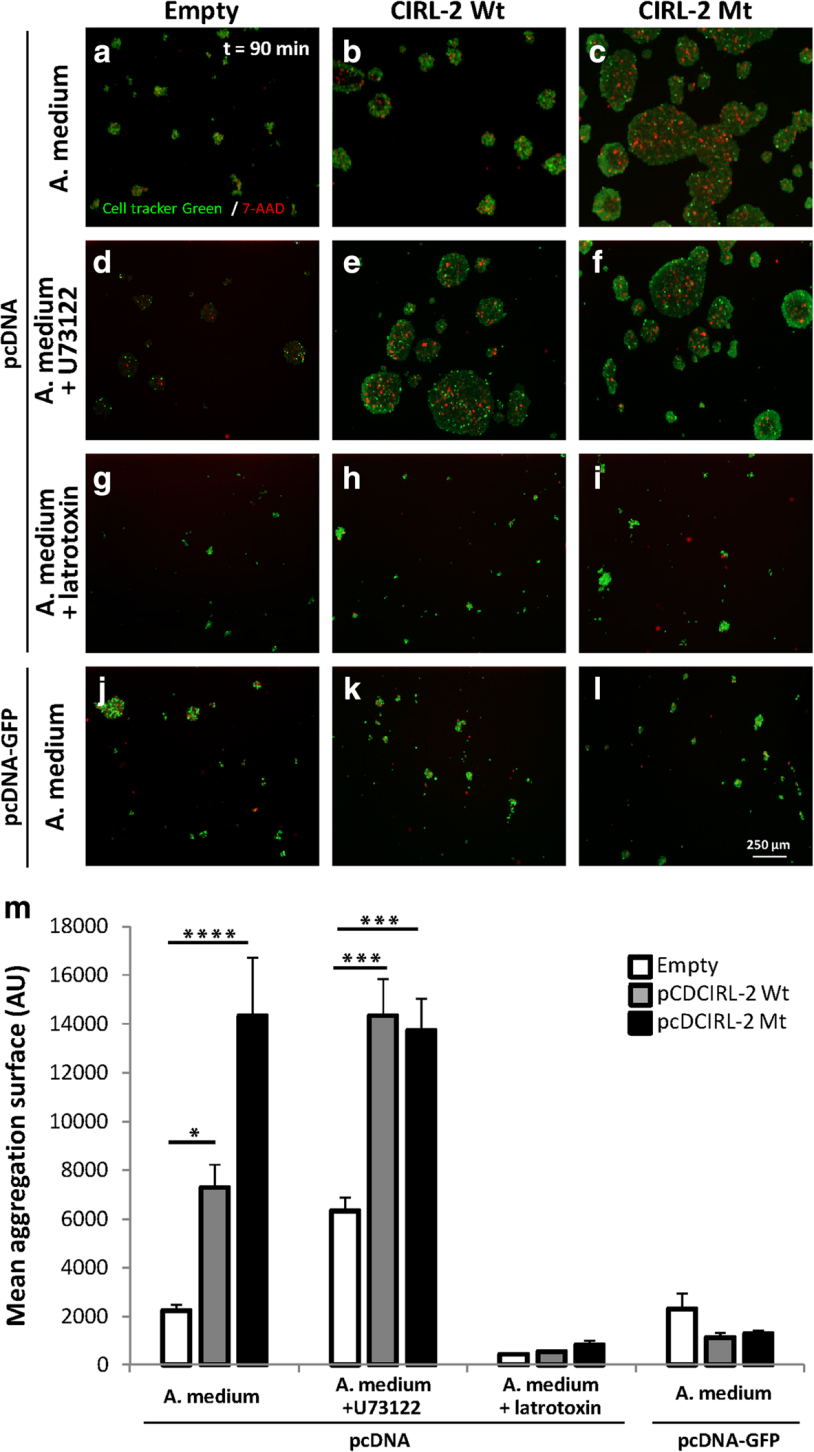
HeLa cells overexpressing mutant *ADGRL2* present enhanced cell adhesive properties associated to signal transduction alteration. **a-c** HeLa cells expressing either pcD-Empty (**a**), CIRL2-Wt (**b**) or CIRL2-Mt (**c**) were labelled with the viability marker, cell tracker green (10 μM), and the mortality marker 7-AAD (50 μg/ml) and incubated at room temperature for 90 min in aggregation medium. Note the marked increase of aggregate sizes in CIRL2-Mt expressing cells. **d-f** HeLa cells expressing either pcD-Empty (**d**) CIRL2-Wt (**e**) or CIRL2-Mt (**f**) were incubated at room temperature for 90 min in aggregation medium containing the PLC inhibitor U73122 (3 μM). Note that inhibition of PLC enhanced homophilic binding of HeLa cells overexpressing CIRL2-Wt. **g-i** HeLa cells expressing either pcD-Empty (**g**), CIRL2-Wt (**h**) or CIRL2-Mt (**i**) were incubated at room temperature for 90 min in aggregation medium containing α-latrotoxin (1 nM) which prevented cell aggregation. **j-l** HeLa cells expressing either pcD-GFP-Empty (**j**), CIRL2-GFP-Wt (**k**) or CRL2-GFP-Mt (**l**) were incubated at room temperature for 90 min in aggregation medium. Cells expressing CIRL2 coupled to GFP in C-terminal were not able to aggregate. **m** Quantification and statistical analysis of the aggregation index. Each value represents the mean (±S.E.M.) of three independent cell-adhesion assays. **, *p* < 0.01; ***, *p* < 0.001, ****, *p < 0.0001* using one-way ANOVA test

Given the close relationship between Ca^2+^_i_ concentration, cell adhesion and migration [36, 37, 74] and because latrophilin function as cell-adhesion molecules [12], an alteration of the ADGRL2 signal transduction could induce modifications in cell adhesive properties. A cell adhesion assay was therefore developed to allow measurement of the binding of cells to each other as a function of Adgrl2 plasma membrane expression in HeLa cells (adapted from Boucard et al. [12], Additional file 7: Figure S4). In line with microfluorimetry experiment results, when pcDCIRL-2 coupled to GFP was overexpressed, no cellular aggregates were observed, suggesting that absence of homophilic cell adhesion properties explained the calcimetry results (Fig. 8j, k, l) [12]. In contrast to pcD-empty plasmid, cells expressing wild-type pcDCIRL-2 assembled to each other to form aggregates after 90 min under gentle stirring, suggesting homophilic cell adhesion (Fig. 8a, b, m). The size of cell aggregates was two times larger for cells overexpressing the mutant pcDCIRL-2 construct (Fig. 8c). As rat Adgrl2 homolog CIRL-2 carrying the variation is unable to transduce any signal in response to its activation, a link between cell adhesion and G protein–mediated intracellular signalling is highly conceivable. To confirm this hypothesis, PLC was blocked by adding 10 μM U73122 in the aggregation medium (Fig. 8d, e, f). Under this condition, aggregate size of cells overexpressing CIRL-2 Wt reached the size of aggregates overexpressing CIRL-2 Mt. (Fig. 8e, f, m). Treatment by U73122 of cells transfected with the empty plasmid also allowed for aggregate size increase (Fig. 8m). The effect on aggregate formation under massive increase of cytosolic Ca^2+^_i_ was also investigated by adding α-latrotoxin in the aggregation medium. After 90 min, no cell aggregates were formed (Fig. 8g-i, m). These findings support that PLC coupling and Ca^2+^_i_ concentrations affect cell adhesion properties, with higher Ca^2+^_i_ concentration being correlated with the lower adhesion.

**Fig. 9:**
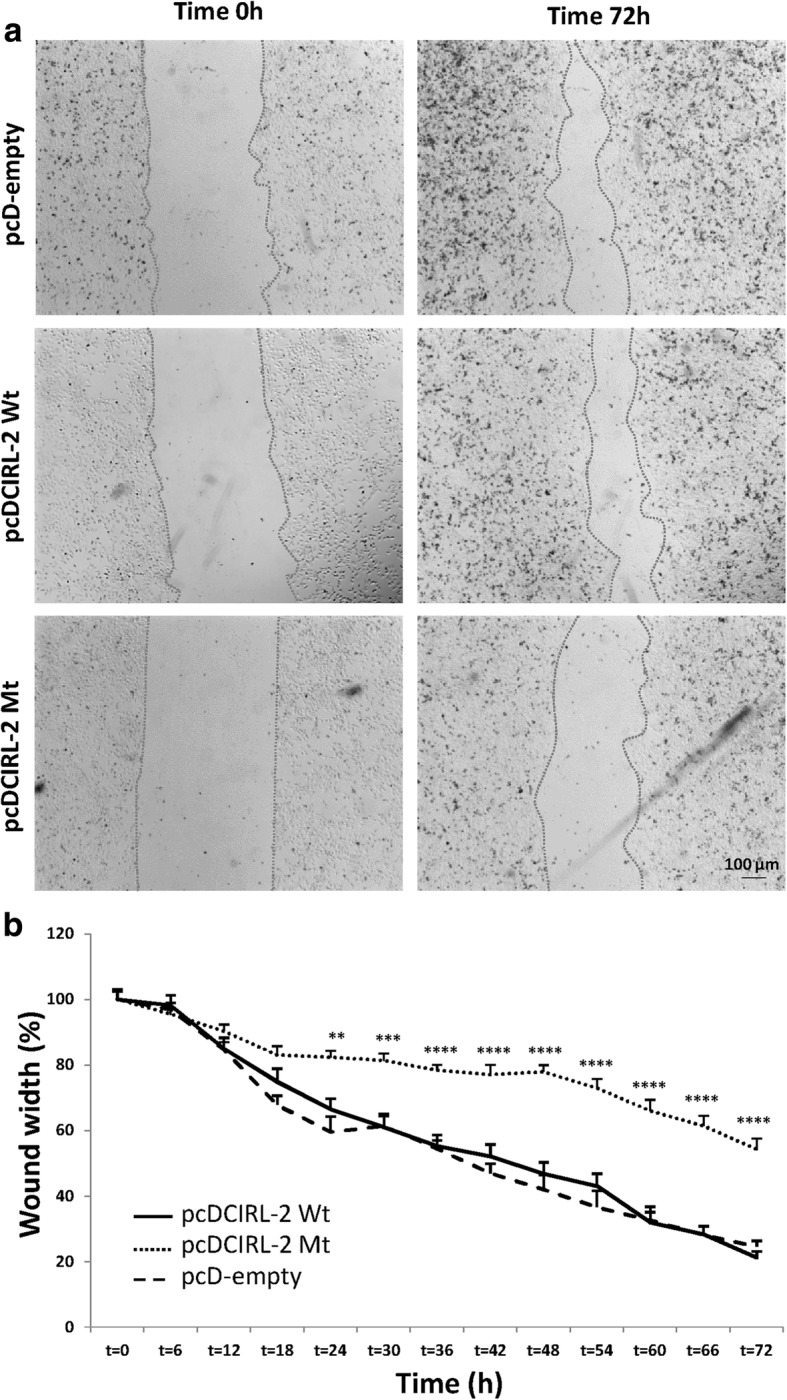
Cell motility is altered in HeLa cells overexpressing mutant ADGRL2. **a** Wound healing experiments were performed on monolayer HeLa cells overexpressing pcD-empty, CIRL2-Wt or CIRL2-Mt. Microphotographs visualize wound healing images acquired 0 and 72 h after the scratch. **b** Time-course quantification of scratch width in monolayer HeLa cells overexpressing pcD-empty, CIRL2-Wt or CIRL2-Mt. Each value represents the mean (±S.E.M.) from three independent experiments. **, *p* < 0.01; ***, *p* < 0.001; ****, *p* < 0.0001 using two-way ANOVA test

To determine if cell adhesion excess was associated with cell motility modulation when *Adgrl2* is overexpressed in HeLa cells, scratch assays were carried out (Fig. 9) [44]. In pcD-empty cells, the wound width was reduced by 56.6% after 72 h of culture. Although no differences were found between pcD-empty and pcDCIRL2-Wt cells, a dramatic reduction of wound healing was observed in cells overexpressing pcDCIRL2-Mt (Fig. 9a, b). To ensure that wound healing inhibition resulted from cell migration or proliferation alteration, cell cycle experiments were performed. Although the PI was evaluated at 48.8%, with 34.4% of cells in S phase in pcD-empty cells, PI was evaluated at 41.5% with 27.7% of cells in S phase in pcDCIRL2-Wt cells and PI was evaluated at 43.1% with 28.3% of cells in S phase in pcDCIRL2-Mt cells respectively. For each condition, the percentage of cells in G2/M phases was not significantly different and was between 13.8 and 15.1%. Cell cycle data were not different among the three transfected cell conditions, strongly suggesting that wound healing inhibition quantified in pcDCIRL2-Mt cells is unlikely to be due to effects on cell division or proliferation, which argues for delayed cell motility and migration due to an over-adhesion mechanism.

### Mutant *ADGRL2* cells present size and cytoskeletal network alterations

One of the mechanisms required for cell adhesion consists in fluctuations of Ca^2+^_i_, which regulate the dynamic assembly of cytoskeletal elements [45, 62]. To visualize F-actin and microtubule network modifications, glass coverslips cultured HeLa cells overexpressing pcD-empty, pcDCIRL-2 Wt or pcDCIRL-2 Mt. were immunolabelled with phalloidin and acetylated α-tubulin (Additional file 8: Figure S5). Depending on the transfected plasmid, cells harboured various morphologies: pcD-empty cells possessed a fusiform shape (Additional file 8: Figure S5a) while pcDCIRL-2 Wt spread out on the substratum (Additional file 8: Figure S5b). In the pcDCIRL-2 Mt. condition, cells were significantly more spread out than pcDCIRL-2 Wt cells (Additional file 8: Figure S5c). However, when detached from their culture support, cells from these three conditions displayed only minor differences concerning their size and content. Using flow cytometry quantitative studies, HeLa cultured cells had a mean size of 234.2 ± 5.95 AU, HeLa cells transfected with an empty vector a mean size of 241.7+/− 8.30 AU, HeLa cells transfected with the Wt *Adgrl2* a mean size of 238.7 ± 6.73 AU and cells transfected with the *ADGRL2* variant a mean value of 258.6 ± 13.49 AU. Regarding intracellular heterogeneity, some differences were observed between HeLa cells alone (236.7 ± 3.65 AU) and cells transfected with the *ADGRL2* variant (264.9 ± 5.70 AU). From these data, increased size of cells overexpressing Adgrl2 (Additional file 8: Figure S5b, c) appears to be directly associated with their over-adhesive properties. Similar to flow cytometry studies, which evidenced intracellular heterogeneity differences among the different conditions, immunohistochemistry revealed cytoskeleton architecture modifications (Additional file 8: Figure S5a-c). In the three conditions, the α-tubulin network was located in the perinuclear area, whereas the F-actin network appeared to be modified in the case of *Adgrl2* overexpression. In control cells, a well-defined F-actin filament network was essentially located at the periphery of the cell (Additional file 8: Figure S5a). When *Adgrl2* was overexpressed, cells displayed a highly developed cytoplasmic F-actin network with numerous anchoring points to the glass coverslip (Additional file 8: Figure S5b; arrows). Overexpression of the mutant *Adgrl2* exacerbated this phenomenon in the cell surface membrane of HeLa cells by homophilic Adgrl2 interaction activation (Additional file 8: Figure S5c). Thus, the *ADGRL2* c.3785T>A;p.(Leu1262His) variant that alters signal transduction coupled to G protein is responsible for excessive adhesion properties associated with abnormal cytoskeletal remodelling of cells overexpressing this variant.

## Discussion

Within the large GPCR superfamily, ADGRL2 (previously named LPHN2) together with ADGRL1 (previously named LPHN1), ADGRL3 (previously named LPHN3) and ADGRL4 (previously named ELTD1) belong to the Adhesion family encompassing 33 mammalian members [26]. Adhesion-GPCRs are involved in several key molecular and cellular functions, including planar cell polarity, regulation of cytoskeleton organization, cell adhesion and migration, cell cycle, cell death and differentiation [26], but the precise mechanisms by which ADGRL2 acts remain elusive. An exogenous agonist for ADGRLs has long been identified: α-latrotoxin, the major neurotoxin in black widow spider venom, which attests a synaptic role for ADGRLs [47]. Endogenous ligands include Teneurin-2 (also known as Lasso), neurexins and FLRT1–3 (Fibronectin Leucine-Rich Transmembrane protein) [11, 48, 54, 63]. Contrary to ADGRL1 and ADGRL3, ADGRL2 does not bind to neurexins (neurexin-1a, −1b, −2b), binds only weakly to teneurin-2, and interacts with FLRT3, but not with FLRT1. Because ADGRLs were first identified as putative synaptic receptors for α-latrotoxin, researchers initially focused their attention on the role of ADGRL2 in the synapse, showing that ADGRL2 could mediate synapse recognition and assembly and/or contribute to a synapse maintenance signal [2].

The c.3785T>A;p.(Leu1262His) variant is localized in the intracellular domain of ADGRL2, which exhibits 35% and 49% similarity between ADGRL1 and ADGRL3 proteins, respectively. G protein–mediated intracellular signalling has been demonstrated for ADGRL1 by its interaction with Gα_o_ and its binding to teneurin-2, which induces Ca^2+^ signals [42, 63]. Microfluorimetry experiments confirmed that the c.3785T>A variant impairs the early stage of cytosolic Ca^2+^_i_ release in response to α-latrotoxin binding, both in the patient’s amniocytes and in HeLa cells transfected with the mutant Adgrl2 construct. Moreover, addition of the PLC inhibitor U73122 in wild-type amniocytes induced early step calcium release impairment. Thus ADGRL2 activation is necessary for G protein–mediated intracellular signalling. More precisely, ADGRL2 is required for this early step of calcium release, whereas α-latrotoxin tetramer pores are responsible for passive Ca^2+^_e_ influx into the cell during the second step. Pure α-latrotoxin is a very stable homodimer, which further assembles into tetramers in the presence of Mg^2+^ or Ca^2+^ to form α-latrotoxin pores [3]. Cyclic nucleotide signalling and Ca^2+^ are known to be intracellular downstream targets for many extracellular guidance molecules. They convert the information from locally expressed guidance molecules to intracellular effectors, which control migration by regulating cytoskeleton dynamics, in particular the F-actin network. Using cerebellar granule cell cultures, Komuro et al. demonstrated that their migration speed from the transient external granule cell layer was correlated to both the amplitude and frequency of Ca^2+^ elevations, and that the reduction of the Ca^2+^ transients resulted in the termination of granule cell migration [37]. The modulation of intracellular Ca^2+^ release by the adhesion-GPCR ADGRL2 could therefore exert a role in the regulation of neuronal migration. Cell adhesion assay confirmed that the inhibition of the G protein–mediated intracellular signalling was correlated with enhanced adhesion and increase in size of aggregates overexpressing mutant *Adgrl2*. Transfection of either wild-type or mutant-type pcDCIRL-2, the rat homologue of *ADGRL2*, in HeLa cells induced a strongly developed microtubule network with many focal adhesion points, indicating that *ADGRL2* inactivation leads to increased adhesion properties due to intracellular Ca^2+^ flux and cytoskeletal organization perturbations. Further characterisation of focal adhesions points could provide additional evidence of cell migration alteration as the recruitment of talin and vinculin is correlated with the mechanical force applied to the focal adhesion [18].

The causal effect of the c.3785T>A;p.(Leu1262His) variation in *ADGRL2* on the fœtal brain malformation in *Adgrl2*^+/−^ mice, *Adgrl2*^−/−^ genotype being lethal in utero at E15.5 was supported by MRI findings. Both male and female *Adgrl2*^+/−^ adult mice displayed microcephaly, affecting mainly the telencephalon, although more severe in *Adgrl2*^+/−^ female mice, with a defect in anteroposterior growth of the vermis, in line with ADGRL2 expression during telencephalic and cerebellar development in human embryos and fœtuses as well as in chicks and mice.

Our results suggest that this *ADGRL2* variation impedes the proper development of the cerebellum resulting in RES which is thought to occur when the cerebellar primordium develops and probably results from abnormal function of genes expressed during initial patterning of the mesencephalon-rhombencephalon [43, 68]. For some authors, RES is thought to result from loss of anterior cerebellar anlage cells derived from the medial ventricular zone (VZ) of the cerebellar primordium destined to become the vermis, or from a shift of these anterior cells toward a more posterior and/or ventral hemispheric fate [56]. For others, RES results rather from a loss of posterior expansion of the medial region of the cerebellar primordium [61]. Expansion of the medial cerebellar primordium plays a considerable role in generating the vermis and the hemispheres, which grow together rapidly from the two other germinal zones, the rhombic lips. Although the precise mechanisms that lead to the proper individualisation of the vermis and the hemispheres (which are lacking in RES) remain unknown, *ADGRL2* variation likely avoids expansion of the vermis and of the hemispheres from the medial VZ and EGL. Besides, the ADGRL2 ligand, FLRT3, has a strong specific expression in the isthmic organizer, whereas ADGRL2 is not expressed in this area [25]. This non-overlapping pattern of expression could be due to a specific dosage code to specify the boundaries of the future cerebellum along the rostrocaudal axis of the rhombencephalon. Interestingly, it has also been shown that lat-1, an orthologue of vertebrate ADGRLs in *C. elegans*, plays an essential role in anterior-posterior tissue polarity in the embryo [41, 51]. Moreover, in the absence of *lat-1*, stem cells display positioning defects as they remain clustered near their place of birth, highly reminiscent of what observed in *ADGRL2* mutant cells using wound healing experiments [41]. Our results suggest that RES could result from abnormal positional cues along the anteroposterior axis.

Primary congenital microcephaly and microlissencephaly have classically been classified as disorders of neurogenesis, consisting above all in a severe decrease of symmetric and asymmetric divisions in the ventricular and subventricular zones resulting in neuronal cell production depletion. These disorders could be attributed to four major pathophysiological mechanisms, including first, early events (during the 5th gestational week) that have been postulated as being of major importance for the control of cell cycle and proper functioning of the primary cilium; second, abnormalities during the different phases of mitosis (prometaphase, metaphase and cytodieresis); third, anomalies of formation and positioning of the mitotic spindle; and fourth, abnormal structure and function of associated molecules that are involved in cytoskeletal dynamics.

A constellation of disease-causing genes have been described as affecting the early steps of neural tube development, i.e., planar polarity, primary cilium structure (such as kinesins) or functions involving in particular Wnt and Sonic hedgehog pathways, as well as cell cycle length controlling molecules, notably *PAX6* and *FLNA* [67].

As regards the different phases of mitosis, *WDR81* deleterious variants were shown to increase the number of mitotic cells with an accumulation of cells in prometaphase and metaphase resulting in mitotic delay without any impact on mitotic spindle or primary cilia organization [13]. Variations in the *CIT* gene encoding citron kinase localizes to the cleavage furrow and midbody, where it functions in cytodieresis. The neuropathological phenotype includes extreme microcephaly or microlissencephaly [28].

In fact, the vast majority of genes responsible for microcephaly encode centrosomal proteins involved in spindle orientation or proteins regulating these centrosomal proteins. To date, bi-allelic deleterious variations in 13 genes (*MCPH1*, *WDR62*, *CDK5RAP2, CASC5*, *ASPM*, *CENPJ*, *STIL, CEP135, CEP152, ZNF335, PHC1, CDK6, CENPE* [20, 57]) which are normally expressed in ventricular (apical) and subventricular (basal) radial glial cells, impair proper spindle positioning and orientation leading to a drastic decrease in the generation of intermediate progenitors and of post-mitotic neuroblasts due to a lack of maintenance of centrosome asymmetry [73]. In these pathological conditions, brain lesions range from microcephaly with simplified gyral pattern to microlissencephaly. Pathogenic variations in *NDE1* which interacts directly with LIS1 and is expressed in the neuroepithelium of the cerebral and cerebellar cortices where it contributes to interkinetic nuclear migration and also localized at the centrosome and on the mitotic spindle, result in early failure of neuron production and microlissencephaly [1, 7].

Microtubule structure alterations as well as alterations of binding partners necessary for proper microtubule dynamics resulting from pathogenic variants in *TUBA1A, TUBB2A, TUBB2B, TUBB3, TUBB5, TUBG1* and *TUBA8* genes are also responsible microcephaly and microlissencephaly. These lesions are constantly associated with several brain abnormalities due to defects in progenitor proliferation with decreased symmetric divisions, impaired neuronal radial migration and abnormal neuronal differentiation resulting in deficient neurite outgrowth, axon path-finding and connectivity. It is worth noting that in all these conditions, various brainstem and cerebellar abnormalities have been described, but RES has never been identified [5, 14].

Other pathophysiological mechanisms that may cause primary microcephaly have been recently identified, among others microRNAs and Golgi trafficking defects [46, 58]. But to our knowledge, microcephaly due to excessive adhesion of progenitors in the germinal zones (VZ, inner SVZ and outer SVZ) has never reported so far.

In the present case, no lesions that could argue for one of the above mentioned pathophysiological mechanisms were identified. Although lat-1, the orthologue of vertebrate ADGRLs in *C. elegans*, is required for cell division plane orientation in the *C. elegans* embryo [41, 51], normal proliferative indices in the LGE indicate that neither symmetric nor asymmetric divisions are altered by comparison with the fœtus harbouring a deleterious variant in the *MCPH1* gene and in which the proliferative index was reduced by a factor of two. Also due to normal proliferative indices, extreme microcephaly with no sulcation associated with this *ADGRL2* variant is unlikely to be connected to mitotic spindle dysfunction. The variation in the *ADGRL2* gene is responsible for Ca^2+^_i_ release alteration that constitutes the step before the modulation of microtubule organization. Furthermore, neuronal cells acquire abnormal adhesion and aggregation properties that make them stay in the subventricular zone and probably thereafter prompt them to die. All these data highlight a new mechanism for microcephaly that results from an excess of cell adhesion of neuronal progenitors in the germinal zones, which could also at least partly explain the co-occurrence of RES.

Unlike *ADGRL2* variations that have never been reported to be responsible for human pathologies, variants in other adhesion-GPCR have been recognized, notably *ADGRG1* (previously named *GPR56*), which is responsible for an autosomal recessive condition associated with the presentation of bilateral fronto-parietal polymicrogyria associated with cerebellar hypoplasia, dysgenesis and pseudo-cysts [6, 59]. Similar to humans, *Adgrg1/Gpr56* null mice display a malformed cerebral cortex resembling cobblestone lissencephaly with similar cerebellar lesions [49]. The rostral cerebellar defects result from specific failure of adhesion of the late migrating granule cells to extracellular molecules of the glia limitans whose structural integrity is disrupted with subsequent overmigration of granule cells into the subarachnoid spaces, but not from intrinsic defects in neuronal proliferation and migration [35]. In contrast to ADGRG1/GPR56, which promotes cell adhesion of developing neurons to basal lamina molecules, ADGRL2 promotes cell migration by controlling Ca^2+^_i_ release.

## Conclusions

In conclusion, we have identified an *ADGRL2* variation very likely responsible for severe microcephaly with almost no sulcation highlighting a new mechanism for the two associated malformations related to excessive adhesion of neuron progenitors within the germinal zones at least mediated by reduction of Ca^2+^ transients. Given its role in the determination of the anteroposterior axis suggested by its orthologue lat-1 [41, 51], we also hypothesize that RES results from abnormal positional cue alterations and defective expansion of the medial VZ in the cerebellar primordium. The identification of ADGRL2 ligands and/or of pathogenic variations in other genes associated with RES will shed more light on the role of ADGRL2 in the pathophysiology of this rare condition. Finally, in addition to its role in synapse assembly, our observations reveal the role of ADGRL2 in development, as already shown for other adhesion GPCRs.

## Additional files

Additional file 1: **Table S1.** Whole Exome Sequencing qualities. For the three exomes performed on Illumina GAIIx (2x76pb) are summarized: the number of sequenced reads, the yield in Gigagabase, the number and the percentage of reads mapped on the human reference sequence (Hg19), the mean depth of the exome, the percentage of base that have been read more than 10 or 50 times, the percentage of bases with a Qscore of at least 30, the mean quality score and the percentage of reads on target captured. (DOCX 16 kb)

Additional file 2: **Table S2.** Age and cause of death in human cases for ADGRL2 Immunohistochemical. (DOCX 16 kb)

Additional file 3: **Table S3.** Statistical analysis (DOCX 23 kb)

Additional file 4: **Figure S1.** ADGRL2 immunoreactivity in the fœtal female and male gonads. a Clustered primordial follicles in the superficial ovarian cortex, containing strongly immunoreactive oocytes (arrow) surrounded by a single layer of flattened granulosa cells at 24WG [OM × 250]. b At birth, some primary follicles are present, with a centrally placed oocytes (arrow head) surrounded by multilayered ADGRL2-positive granulosa cells (thick arrow) [OM × 400] with weaker immunoreactivity of interstitial cells. c Numerous Leydig cells (arrow) being positive in the ovarian hilum at 36WG [OM × 400]. d Multiple seminiferous tubules composed of moderately immunoreactive Sertoli cells (arrow head) and strongly immunolabelled spermatogonia (thick arrow) in a testis at 32WG. Interstitial Leydig and mesenchymal cells are also moderately immunoreactive (asterisk) [OM × 100]. (TIF 22156 kb)

Additional file 5: **Figure S2.** Expression of ADGRL2 in patient amniocytes and control amniocytes cells. a Western blot analyses of amniocytes cells lysates obtained from patient (P) and two control fœtuses (C1 and C2) of the same development stage. Blot was probed with an antibody that recognizes ADGRL2 or GAPDH protein (loading control). Anti-ADGRL2 antibody recognizes two forms of ADGRL2: 163 kDa (precursor) and 72 kDa (N-terminal fragment). b Quantification of ADGRL2 precursor and N-terminal fragments was performed using GAPDH as the loading control. The histogram represents mean values (±S.E.M.) of three independent experiences. (TIF 7407 kb)

Additional file 6: **Figure S3.** Signal transduction coupled to G protein is altered in HeLa cell overexpressing mutant *ADGRL2*. a, b Confocal fluorescence image of GFP-tagged CIRL-2 in transfected HeLa cells (a). Nuclei are labelled with Hœchst (b). CIRL2-GFP is expressed as a membrane protein in HeLa cells. c Intracellular calcium was monitored by microfluorimetry of Fura-2 loaded HeLa cells overexpressing wild-type or mutant pcDCIRL-2. Results are expressed as a mean fluorescence intensity (MFI) during time. Alpha-latrotoxin was applied (1 nM) to HeLa cells under calcium free conditions. Three minutes after treatment, extracellular calcium was added. d Quantification of the areas under the curves (UAC, arbitrary units) obtained by the measurement of intracellular calcium levels for the early and late phases in response to α-latrotoxin stimulation. Each value represents the mean (±S.E.M.) of 30 cells. (**, *p* < 0.001 vs Wt using the unpaired t test). (TIF 16017 kb)

Additional file 7: **Figure S4.** Mean aggregation index calculation. a, b For example, cells overexpressing pcDCIRL-2 Mt. were spotted onto culture slides after 0 (a) and 90 min (b) under gentle stirring in aggregation medium. Viable cells were labelled with cell tracker green (green) and dead cells with 7-AAD (red) to control cell viability. c The extent of cell aggregation was assessed by fluorescence microscopy and the resulting images were then analysed by quantifying the number and size of aggregates in the field. Practically, a basal aggregate size was determined on negative control condition and was set as a threshold for image segmentation. The mean aggregation index was calculated using this formula: (sum of aggregate areas / aggregate number)_T90_ − (sum of aggregate areas / aggregate number)_T0_. Scale bar = 250 μm. (TIF 5261 kb)

Additional file 8: **Figure S5.** Cytoskeletal organization is altered in HeLa cells overexpressing mutant ADGRL2. Seventy two hours after transfection, HeLa cells were processed for histochemistry using phalloidin conjugates for F-actin labelling (red), alpha-tubulin antibody (green) and Hœscht as a nucleic acid stain (blue). a HeLa cells overexpressing the pcD-empty plasmid present predominant fusiform shapes with few focal contacts. b HeLa cells overexpressing Wt pcDCIRL-2 are characterized by spread out cytoplasms with numerous focal contacts (arrows). c HeLa cells overexpressing Mt. pcDCIRL-2 present very large and spread out cytoplasms with a very high density of focal contacts (arrows). (TIF 7878 kb)
