# Supplementary material for: A de novo variant in ADGRL2 suggests a novel mechanism underlying the previously undescribed association of extreme microcephaly with severely reduced sulcation and rhombencephalosynapsis
Source: Acta Neuropathol Commun. 2018 Oct 19;6:109. doi: 10.1186/s40478-018-0610-5 (PMC6195752; doi:10.1186/s40478-018-0610-5)
Supplement: Supplementary file 1 — Table S1. Whole Exome Sequencing qualities. For the three exomes performed on Illumina GAIIx (2x76pb) are summarized: the number of sequenced reads, the yield in Gigagabase, the number and the percentage of reads mapped on the human reference sequence (Hg19), the mean depth of the exome, the percentage of base that have been read more than 10 or 50 times, the percentage of bases with a Qscore of at least 30, the mean quality score and the percentage of reads on target captured. (DOCX 16 kb) [file 40478_2018_610_MOESM1_ESM.docx]

**Additional file 1: Table S1** Whole Exome Sequencing qualities. For the three exomes performed on Illumina GAIIx (2x76pb) are summarized: the number of sequenced reads, the yield in Gigagabase, the number and the percentage of reads mapped on the human reference sequence (Hg19), the mean depth of the exome, the percentage of base that have been read more than 10 or 50 times, the percentage of bases with a Qscore of at least 30, the mean quality score and the percentage of reads on target captured.

|  | Fetus | Mother | Father |
| --- | --- | --- | --- |
| Reads | 76625686 | 84606316 | 63934856 |
| Yield (Gb) | 5.7 | 6.3 | 4.8 |
| Reads Mapped | 74510351 | 83126211 | 62001509 |
| % reads mapped | 97.2 | 98.3 | 97 |
| Mean depth | 61.5 | 68.3 | 52.7 |
| % bases 10X | 89.8 | 90.5 | 88.2 |
| % bases 50X | 48.5 | 54.3 | 41 |
| % bases >=Q30 | 89.2 | 89.6 | 88.1 |
| Mean quality score | 35.5 | 35.6 | 35.1 |
| % OnTarget | 65.7 | 65.3 | 67.4 |
