# Supplementary material for: A de novo variant in ADGRL2 suggests a novel mechanism underlying the previously undescribed association of extreme microcephaly with severely reduced sulcation and rhombencephalosynapsis
Source: Acta Neuropathol Commun. 2018 Oct 19;6:109. doi: 10.1186/s40478-018-0610-5 (PMC6195752; doi:10.1186/s40478-018-0610-5)
Supplement: Supplementary file 2 — Table S2. Age and cause of death in human cases for ADGRL2 Immunohistochemical. (DOCX 16 kb) [file 40478_2018_610_MOESM2_ESM.docx]

**Additional file 2: Table S2** Age and cause of death in human cases for ADGRL2 Immunohistochemical.

| Case number | Age | Cerebral maturation* | Medical termination of pregnancy | Cause of death |
| --- | --- | --- | --- | --- |
| 1 | 6 PCW | 6 PCW | No | Unknown |
| 2 | 7 PCW | 7 PCW | No | Unknown |
| 3 | 9 PCW | 10 PCW | No | Spontaneous abortion |
| 4 | 13WG | 13WG | No | Spontaneous abortion |
| 5 | 14WG | 14WG | No | Spontaneous abortion |
| 6 | 16WG | 16WG | No | Premature rupture of membranes  Acute chorioamnionitis |
| 7 | 17WG | 16WG | Yes | Fetal hydrops, unknown cause |
| 8 | 18WG | 16WG | No | Spontaneous abortion |
| 9 | 18WG | 18WG | No | Premature rupture of membranes |
| 12 | 22WG | 22WG | No | Acute chorioamnionitis |
| 13 | 24WG | 24WG | No | Premature rupture of membranes  Acute chorioamnionitis |
| 14 | 25WG | 25WG | No | Premature delivery, dead at birth |
| 15 | 25WG | 25WG | No | Cardiac arrest at birth |
| 16 | 30WG | 30WG | No | Premature delivery  Dead at D6, bilateral adrenal haemorrhage |
| 17 | 32WG | 32WG | No | Dichorial twin pregnancy  Premature delivery dead at birth |
| 18 | 32WG | 34WG | No | Neonatal death, Meconial ileus |
| 19 | 34WG | 34WG | Yes | Fetal hydrops  Viral infection |
| 20 | 34WG | 34WG | Yes | Micromelia |
| 21 | 40WG | 40WG | No | Amniotic fluid inhalation  Dead at H6 |
| 22 | 40WG | 40WG | No | Per partum death  Amniotic fluid inhalation |

PCW: post-conception weeks

WG: weeks of gestation

* according to the morphometric criteria (13WG onward) of Guihard-Costa and Larroche, 1990 [23].
