# Supplementary material for: A de novo variant in ADGRL2 suggests a novel mechanism underlying the previously undescribed association of extreme microcephaly with severely reduced sulcation and rhombencephalosynapsis
Source: Acta Neuropathol Commun. 2018 Oct 19;6:109. doi: 10.1186/s40478-018-0610-5 (PMC6195752; doi:10.1186/s40478-018-0610-5)
Supplement: Supplementary file 3 — Table S3. Statistical analysis (DOCX 23 kb) [file 40478_2018_610_MOESM3_ESM.docx]

**Additional file 3: Table S3** Statistical analysis

| **Experiment** | **Statistical**  **test** | **n** | **Comparison** | ***p<0.05**  ****p<0.01**  *****p<0.001**  ******p<0.0001** |
| --- | --- | --- | --- | --- |
| Calcium microfluorimetry during early phase (Fig 6 d, left panel) | One way ANOVA  followed by a  Tukey's multiple comparisons test (*compared to Wt amniocytes) | (n=5)  5 independent experiments and more than 6 cells analyzed per experiment | ANOVA  Tukey's multiple comparisons test  Wt amniocytes *vs* Mt amniocytes  Wt amniocytes *vs* Wt amniocytes + U73122  Wt amniocytes *vs* Mt amniocytes + U73122 | F=21.84  ****p<0.0001  **p=0.0012  ****p<0.0001  ****p<0.0001 |
| Calcium microfluorimetry on pcDCIRL during early phase (Suppl Fig 3 d) | Unpaired t test | (n=5)  5 independent experiments and more than 6 cells analyzed per experiment | pcDCIRL-2 Wt *vs* pcDCIRL-2 Mt | *p=0.02 |
| MRI analysis of brain volumes (♂+♀)  (body text p17) | Unpaired t test | Wt mice (n=8) Adgrl2^+/-^ mice (n=7) | Wild type mice vs Adgrl2+/- mice | ***p<0.001 |
| MRI analysis of vermis median sagittal planes (Fig 7g,h) | Unpaired t test | Wt mice (n=8) Adgrl2^+/-^ mice (n=7) | Mid-sagittal antero-posterior diameter (mm) ♂ (Fig 5g)  Mid-sagittal antero-posterior diameter (mm) ♀ (Fig 5g)  Mid-sagittal area (mm²) ♀ (Fig 5h) | *p=0.0417  *p=0.0467  *p=0.048 |
| Cell adhesion properties and cell motility (Fig 8 m) | One-way ANOVA  followed by a  Tukey's multiple comparisons test (*compared to empty condition) | n=3 independent experiments | ANOVA  Tukey's multiple comparisons test  ***A. Medium condition***  Empty *vs* pCDCIRL-2 Wt  Empty vs pCDCIRL-2 Mt  ***A. Medium + U73122 condition***  Empty *vs* pCDCIRL-2 Wt  Empty vs pCDCIRL-2 Mt | F= 33.53  ****p<0.0001  *p=0.0483  ****p<0.0001  ***p=0.0003  ***p=0.0009 |
| Scratch width experiments  (Fig 9b) | Two-way ANOVA followed by a  Dunnett's multiple comparisons test  (*compared to pcDCIRL-2 Wt cells) | (n=3)  3 independent experiments and more than 10 measures per experiment | Two-way ANOVA  Dunnett's multiple comparisons test  (*vs* pcDCIRL-2 Mt)  t=0  t=6  t=12  t=18  t=24  t=30  t=36  t=42  t=48  t=54  t=60  t=66  t=72 | Interaction F (36. 52) = 4.657  ****p<0.0001  ns, p> 0.9999  ns, p=0.8854  ns, p=0.5183  ns, p=0.1948  **p=0.0027  *** p=0,0001  ****p<0.0001  ****p<0.0001  ****p<0.0001  ****p<0.0001  ****p<0.0001  ****p<0.0001  ****p<0.0001 |
