# Supplementary figures and images for: A de novo variant in ADGRL2 suggests a novel mechanism underlying the previously undescribed association of extreme microcephaly with severely reduced sulcation and rhombencephalosynapsis
Source: Acta Neuropathol Commun. 2018 Oct 19;6:109. doi: 10.1186/s40478-018-0610-5 (PMC6195752; doi:10.1186/s40478-018-0610-5)

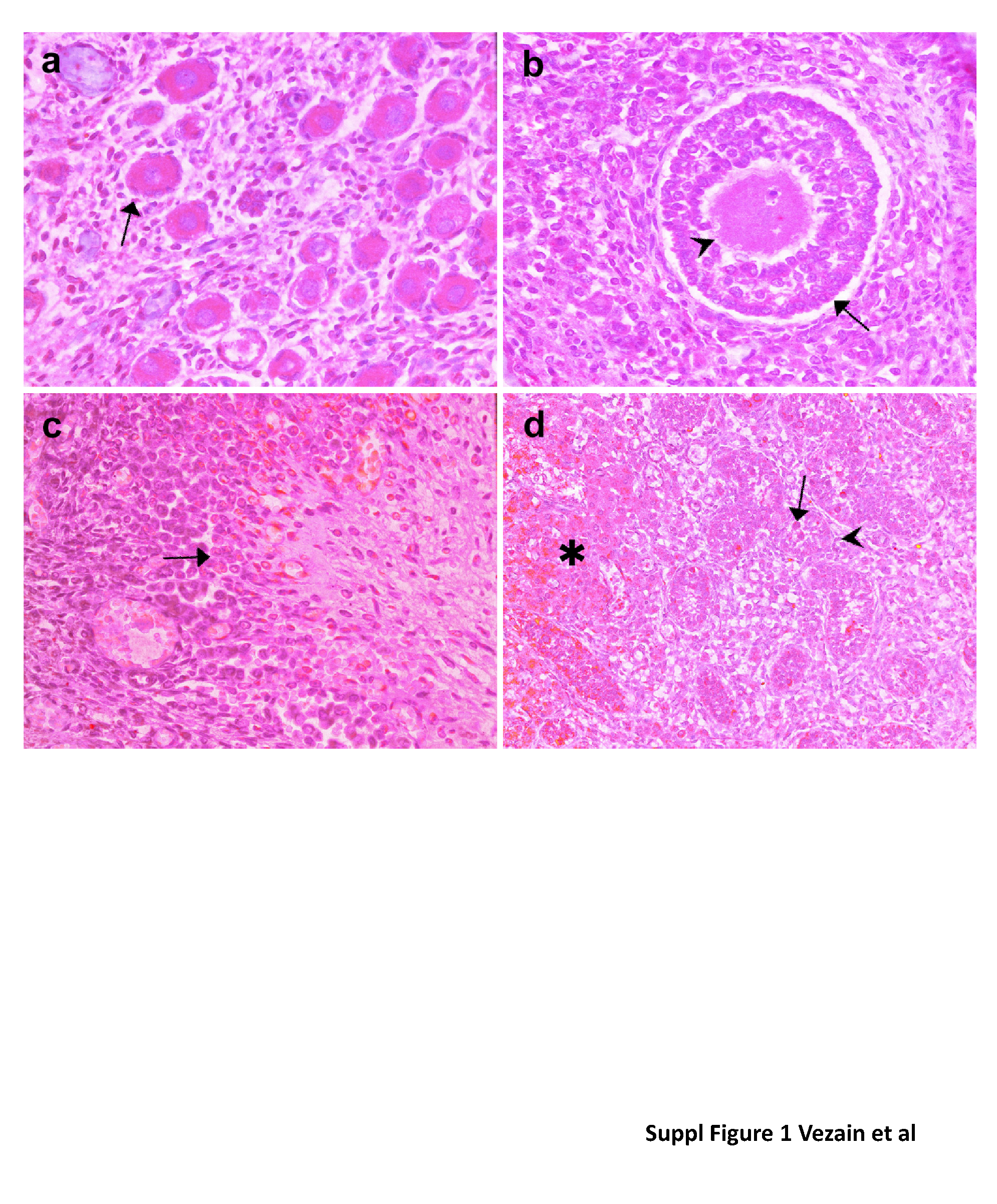

Supplement: Supplementary file 4 — Figure S1. ADGRL2 immunoreactivity in the fœtal female and male gonads. a Clustered primordial follicles in the superficial ovarian cortex, containing strongly immunoreactive oocytes (arrow) surrounded by a single layer of flattened granulosa cells at 24WG [OM × 250]. b At birth, some primary follicles are present, with a centrally placed oocytes (arrow head) surrounded by multilayered ADGRL2-positive granulosa cells (thick arrow) [OM × 400] with weaker immunoreactivity of interstitial cells. c Numerous Leydig cells (arrow) being positive in the ovarian hilum at 36WG [OM × 400]. d Multiple seminiferous tubules composed of moderately immunoreactive Sertoli cells (arrow head) and strongly immunolabelled spermatogonia (thick arrow) in a testis at 32WG. Interstitial Leydig and mesenchymal cells are also moderately immunoreactive (asterisk) [OM × 100]. (TIF 22156 kb) [file 40478_2018_610_MOESM4_ESM.tif]

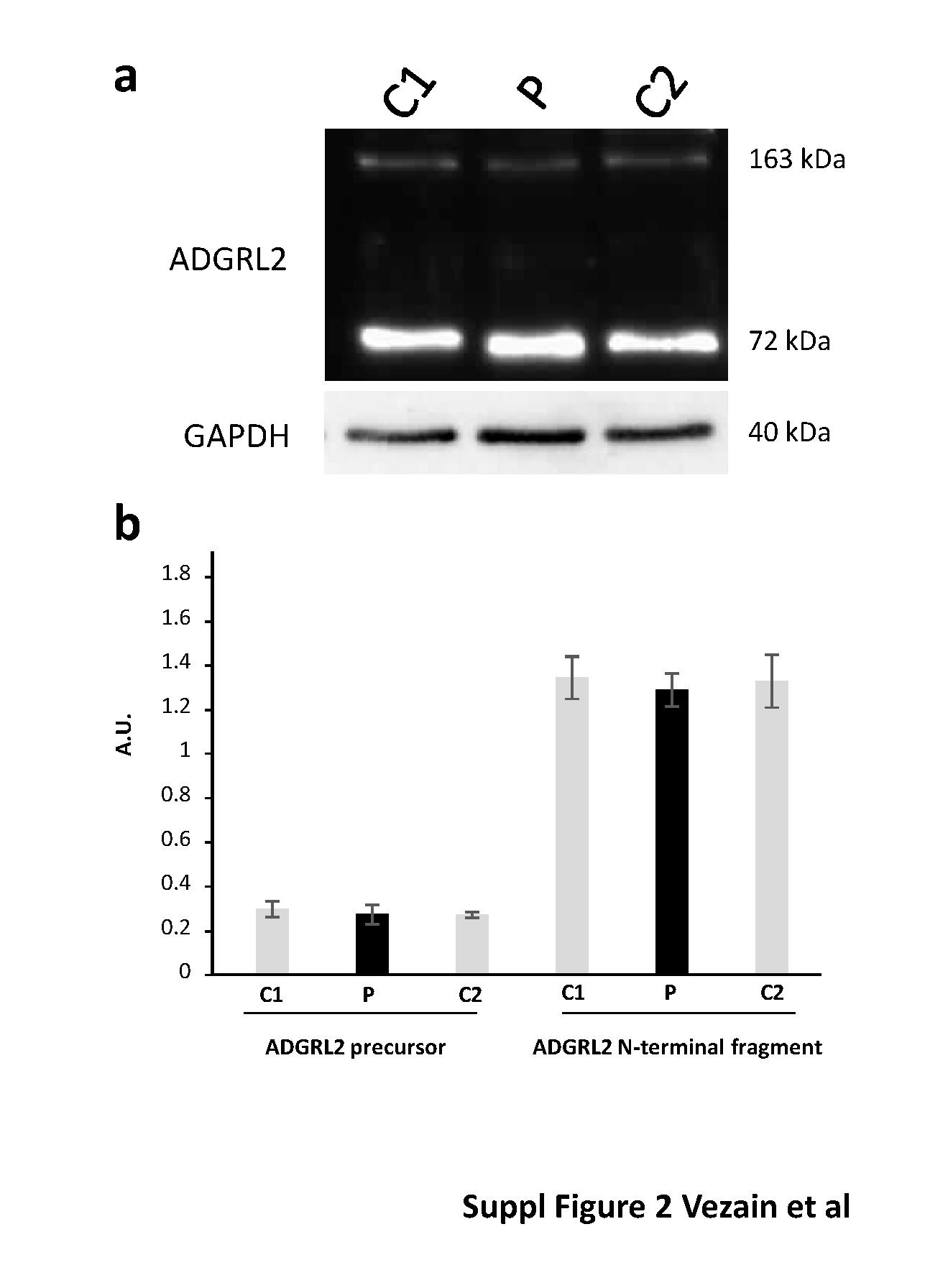

Supplement: Supplementary file 5 — Figure S2. Expression of ADGRL2 in patient amniocytes and control amniocytes cells. a Western blot analyses of amniocytes cells lysates obtained from patient (P) and two control fœtuses (C1 and C2) of the same development stage. Blot was probed with an antibody that recognizes ADGRL2 or GAPDH protein (loading control). Anti-ADGRL2 antibody recognizes two forms of ADGRL2: 163 kDa (precursor) and 72 kDa (N-terminal fragment). b Quantification of ADGRL2 precursor and N-terminal fragments was performed using GAPDH as the loading control. The histogram represents mean values (±S.E.M.) of three independent experiences. (TIF 7407 kb) [file 40478_2018_610_MOESM5_ESM.tif]

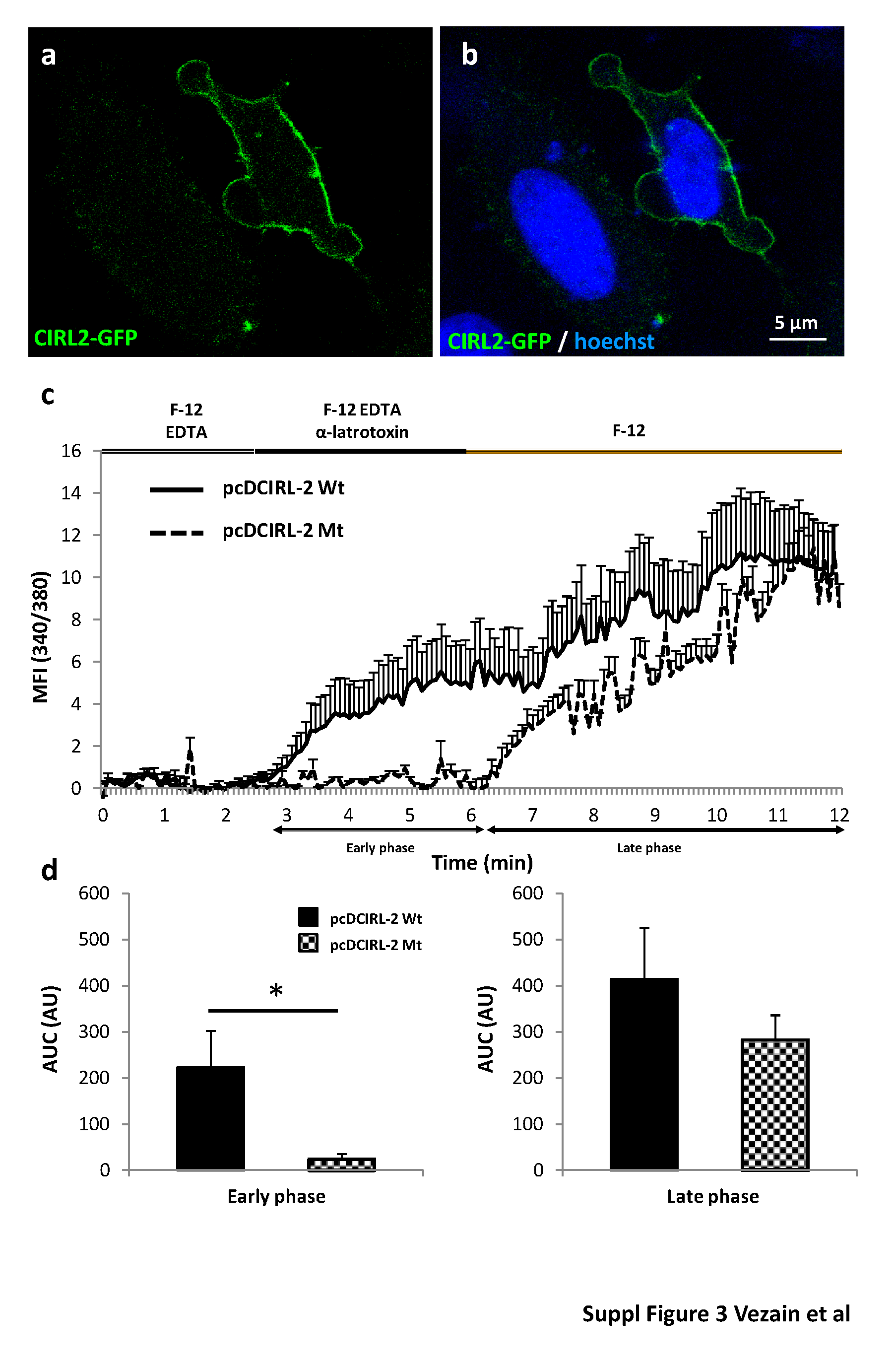

Supplement: Supplementary file 6 — Figure S3. Signal transduction coupled to G protein is altered in HeLa cell overexpressing mutant ADGRL2. a, b Confocal fluorescence image of GFP-tagged CIRL-2 in transfected HeLa cells (a). Nuclei are labelled with Hœchst (b). CIRL2-GFP is expressed as a membrane protein in HeLa cells. c Intracellular calcium was monitored by microfluorimetry of Fura-2 loaded HeLa cells overexpressing wild-type or mutant pcDCIRL-2. Results are expressed as a mean fluorescence intensity (MFI) during time. Alpha-latrotoxin was applied (1 nM) to HeLa cells under calcium free conditions. Three minutes after treatment, extracellular calcium was added. d Quantification of the areas under the curves (UAC, arbitrary units) obtained by the measurement of intracellular calcium levels for the early and late phases in response to α-latrotoxin stimulation. Each value represents the mean (±S.E.M.) of 30 cells. (**, p < 0.001 vs Wt using the unpaired t test). (TIF 16017 kb) [file 40478_2018_610_MOESM6_ESM.tif]

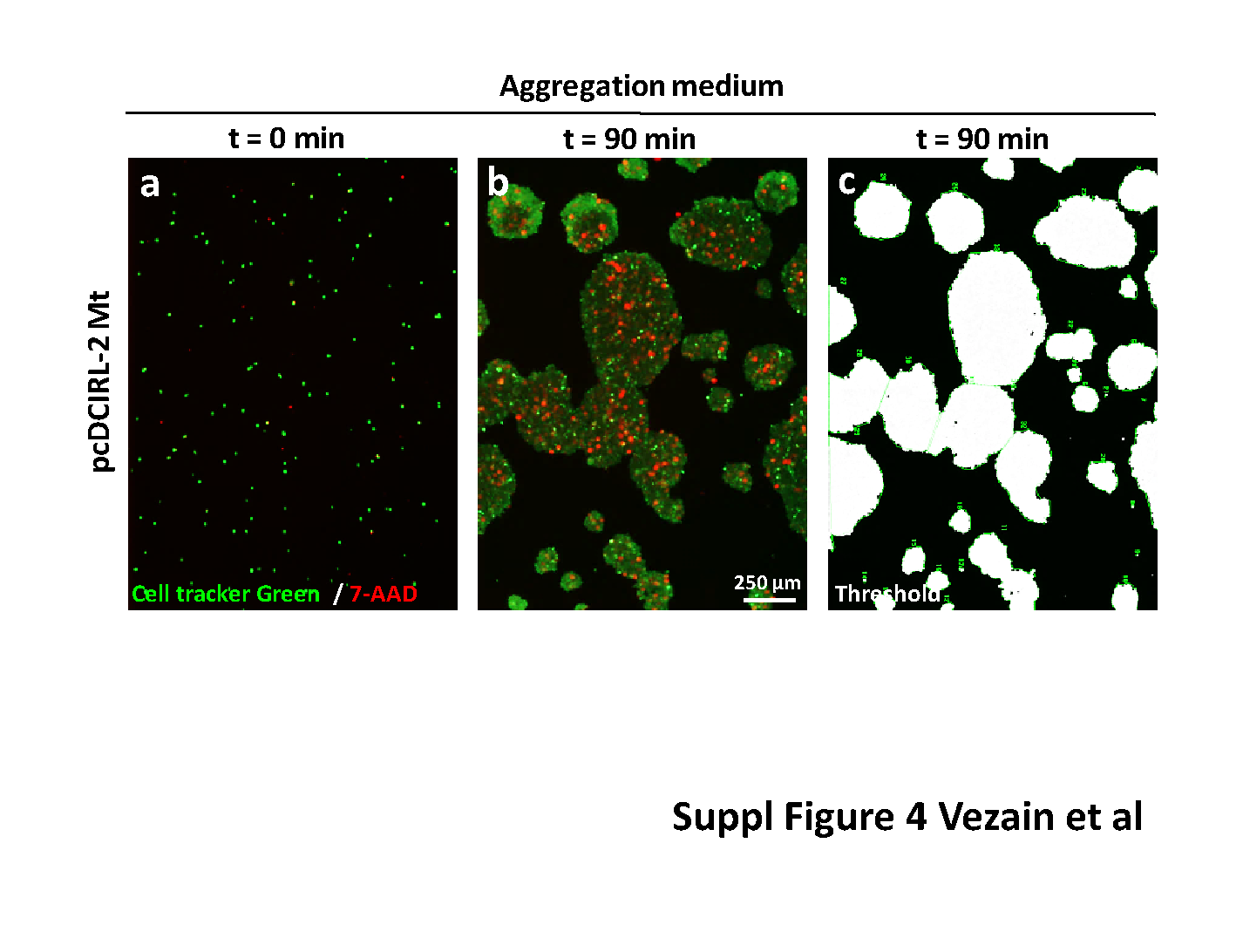

Supplement: Supplementary file 7 — Figure S4. Mean aggregation index calculation. a, b For example, cells overexpressing pcDCIRL-2 Mt. were spotted onto culture slides after 0 (a) and 90 min (b) under gentle stirring in aggregation medium. Viable cells were labelled with cell tracker green (green) and dead cells with 7-AAD (red) to control cell viability. c The extent of cell aggregation was assessed by fluorescence microscopy and the resulting images were then analysed by quantifying the number and size of aggregates in the field. Practically, a basal aggregate size was determined on negative control condition and was set as a threshold for image segmentation. The mean aggregation index was calculated using this formula: (sum of aggregate areas / aggregate number)T90 − (sum of aggregate areas / aggregate number)T0. Scale bar = 250 μm. (TIF 5261 kb) [file 40478_2018_610_MOESM7_ESM.tif]

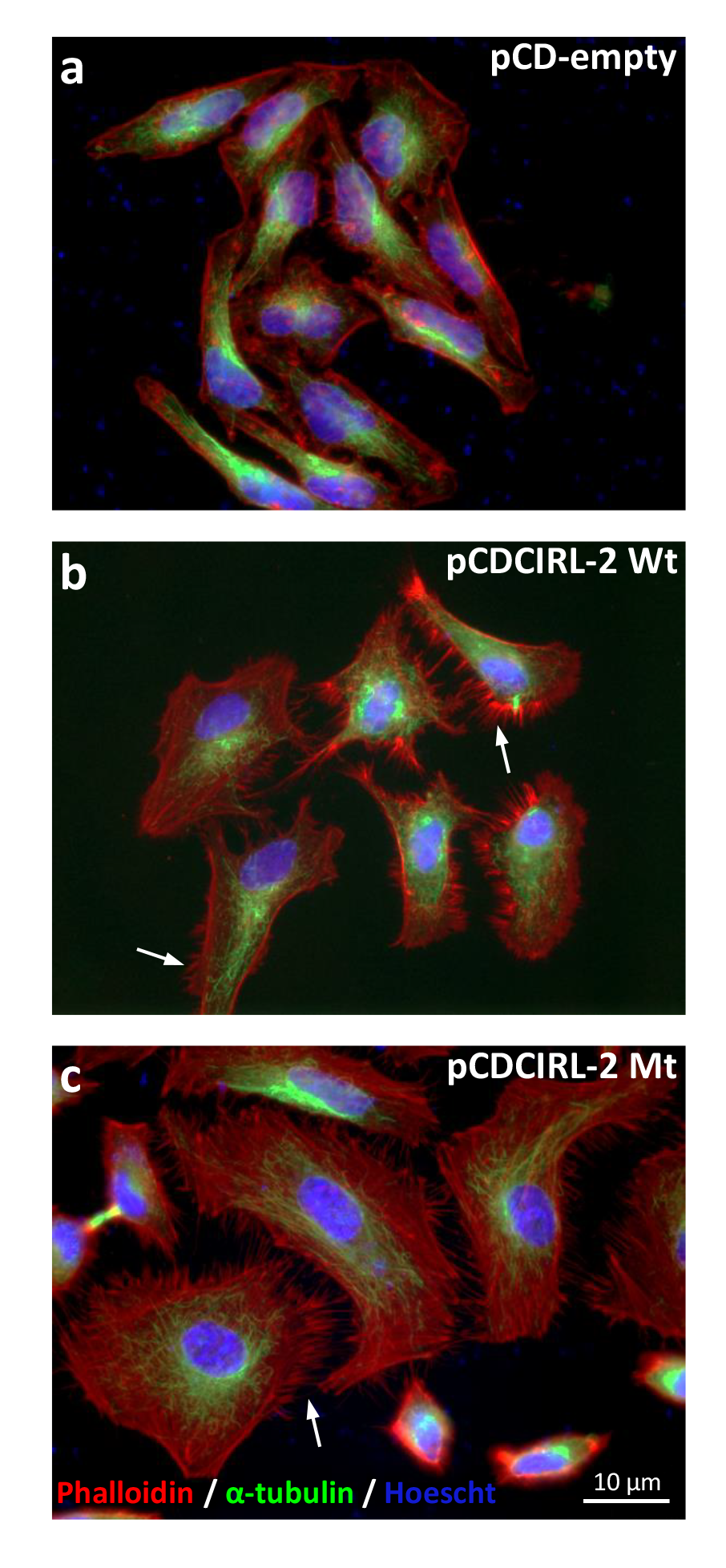

Supplement: Supplementary file 8 — Figure S5. Cytoskeletal organization is altered in HeLa cells overexpressing mutant ADGRL2. Seventy two hours after transfection, HeLa cells were processed for histochemistry using phalloidin conjugates for F-actin labelling (red), alpha-tubulin antibody (green) and Hœscht as a nucleic acid stain (blue). a HeLa cells overexpressing the pcD-empty plasmid present predominant fusiform shapes with few focal contacts. b HeLa cells overexpressing Wt pcDCIRL-2 are characterized by spread out cytoplasms with numerous focal contacts (arrows). c HeLa cells overexpressing Mt. pcDCIRL-2 present very large and spread out cytoplasms with a very high density of focal contacts (arrows). (TIF 7878 kb) [file 40478_2018_610_MOESM8_ESM.tif]
